# Continuous blueshift of self-trapped exciton emission upon compression and decompression in one-dimensional (C_4_H_8_N)_2_PbCl_4_

**DOI:** 10.1039/d6sc04557j

**Published:** 2026-08-15

**Authors:** Peijie Zhang, Xiaofan Xu, Yawen Li, Jiang Han, Yiyang Wang, Zewei Quan

**Affiliations:** a Department of Chemistry, Southern University of Science and Technology (SUSTech) Shenzhen Guangdong 518055 China quanzw@sustech.edu.cn.; b Department of Heterostructured Materials, Interdisciplinary Research Center, Liaoning Academy of Materials Shenyang Liaoning 110170 China

## Abstract

Pressure engineering is a powerful approach for tuning the properties of materials, yet the induced responses are typically reversible. Here, we demonstrate a rare counterexample in the 1D hybrid metal halide (C_4_H_8_N)_2_PbCl_4_, where self-trapped exciton emission exhibits irreversibility upon compression and decompression. The irreversible emission-energy increase of 0.40 eV arises from distinct governing mechanisms along the two pressure pathways. During compression, the blueshift is dominated by a reduction in lattice distortion energy, whereas upon decompression it is sustained by a persistently widened bandgap and a recovery of exciton binding energy. These effects originate from pressure-driven structural modifications of the inorganic [PbCl_4_]^2−^ chains, cooperatively coupled with reactions of the confined organic cations. By revealing an unconventional pathway that couples exciton dynamics with lattice reconstruction and organic chemical reactivity, this work demonstrates that pressure engineering can transcend simple lattice compression and provides a viable strategy for achieving irreversible optoelectronic modulation in hybrid materials.

## Abstract 2

Pressure-induced polyhedral distortion and organic-cation reactions drive an asymmetric compression–decompression pathway in 1D (C_4_H_8_N)_2_PbCl_4_, resulting in an irreversible blueshift of self-trapped exciton emission.

## Graphical abstract

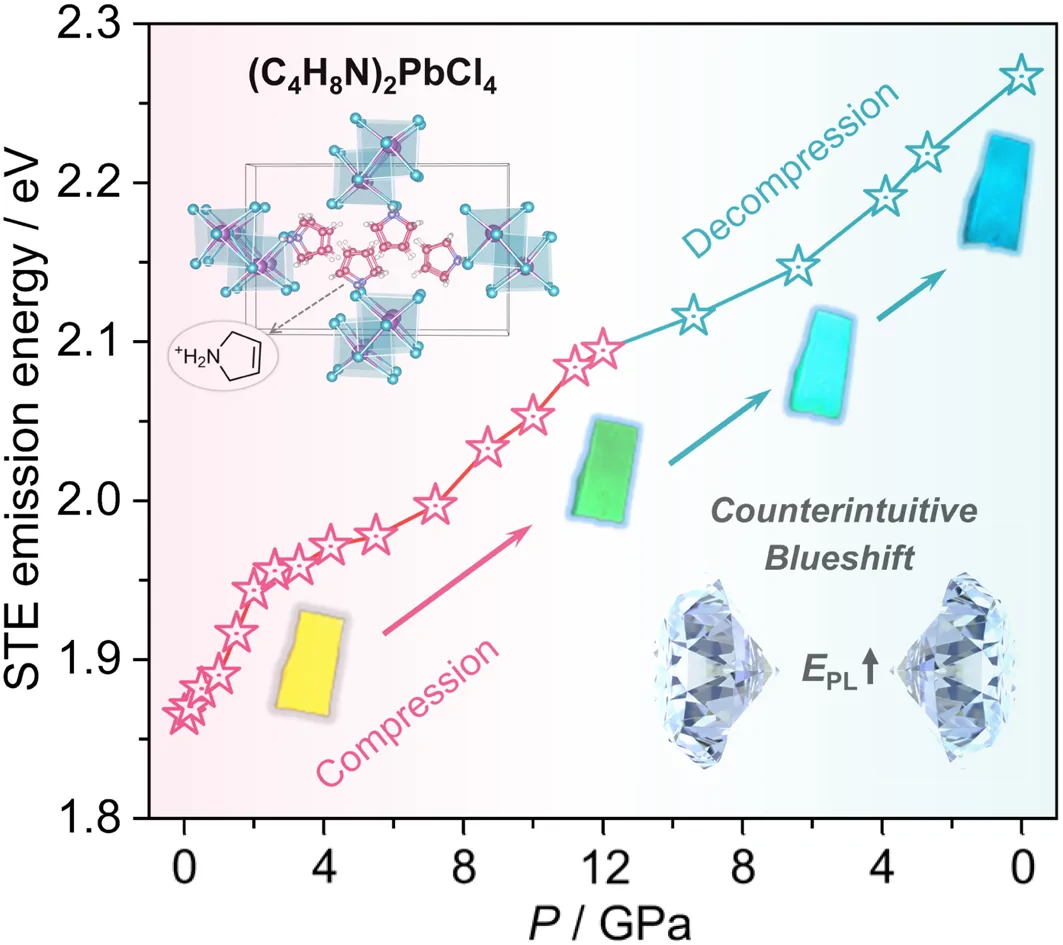

## Introduction

Pressure is a fundamental thermodynamic variable that provides a clean and powerful means to modulate interatomic distances, electronic structures, and lattice dynamics in solids, thereby enabling access to emergent physical and chemical phenomena inaccessible under ambient conditions.^1,2^ In most materials, however, pressure-induced modifications—such as excitonic transitions in WSe_2_–MoSe_2_ heterostructures under 1 GPa or near-room-temperature superconductivity in LaH_10_ above 190 GPa,^3,4^ are reversible once the external pressure is released. This limits the usefulness of high-pressure chemistry for permanent materials design. To move beyond this limitation, recent studies have revealed that pressure can induce genuinely irreversible responses in solids, including pressure-quenched superconducting phases,^5^ pressure aging in ferroelectrics,^6^ and permanent modifications of local chemical bonding, such as pressure-induced polymerization^7–9^ and conformational locking.^10^ These findings collectively indicate that the reversibility of pressure-driven structural evolution is not intrinsic, but instead governed by a delicate interplay between external conditions (*e.g.*, temperature, and pressure) and internal factors (*e.g.*, local bonding environments and size effects).^11^ When this balance is disrupted, pressure can steer materials into metastable states with locked-in structures and emergent functionalities.

Among the diverse classes of functional materials, hybrid metal halides (HMHs) stand out due to their valuable properties, including tunable bandgap, strong defect tolerance, abundant exciton emission, and a combination of chirality.^12–16^ These functionalities originate from their intrinsically soft lattice, pronounced electron–phonon coupling, and high structural flexibility, which collectively make HMHs sensitive to external stimuli. Pressure engineering has emerged as a particularly effective approach for modulating HMHs, as it continuously alters interatomic distances and lattice distortions without altering chemical composition. As a result, high-pressure studies have provided valuable insights into the structure–property relationships of HMHs and have further guided the design of HMHs in the synthesis under ambient conditions.^17–21^ A hallmark photophysical response of HMHs under pressure is their self-trapped exciton (STE) emission. Owing to strong electron–phonon coupling in a soft lattice framework, photoexcited carriers readily induce local lattice distortions, leading to the formation of STEs. This process gives rise to broadband, strongly Stokes-shifted luminescence with high color tunability. Under compression, the STE emission exhibits pronounced and controllable variations in energy, intensity, and excitonic dynamics,^20–23^ providing a sensitive probe for the interplay between local lattice distortions and excited-state behavior. Nevertheless, despite these advantages, most pressure-induced structural and photophysical responses in HMHs follow symmetric and reversible thermodynamic transformation pathways during compression and decompression. Even when pressure irreversibly amorphizes HMHs, the resulting deep-level defects and enhanced nonradiative decay pathways severely quench fluorescence.^24^

In this work, we demonstrate that the one-dimensional (1D) HMH (C_4_H_8_N)_2_PbCl_4_ exhibits anomalous and irreversible STE emission under a compression–decompression cycle. Pressure engineering finally drives the system into a metastable phase with an irreversible enhancement of STE emission energy (*E*_PL_) by 0.40 eV and markedly enhanced luminescence intensity. During compression, the emission blueshift (0.23 eV) arises predominantly from a reduction in lattice deformation energy (*E*_d_). Conversely, during decompression, the increase in bandgap (*E*_g_) and the decrease in exciton binding energy (*E*_b_) both contribute to the blueshift (0.17 eV) for STE. Structural analysis indicates that under compression the system undergoes a phase transformation, while the subsequent pressure release induces enhanced structural disorder, ultimately stabilizing a metastable phase with disordered [PbCl_4_]^2−^ chains. This sequence of structural evolution is likely associated with the reversible reactions of C=C bonds of 3-pyrrolinium cations within the confined environment. Such pressure-driven organic sublattice reactions can drive HMH away from the reversible thermodynamic path in pressure engineering, thus entering a metastable state under the kinetic path. Our findings establish that pressure engineering, by coupling lattice reconstruction with chemical reactivity, can enable irreversible optoelectronic modulations in hybrid materials beyond conventional compression.

## Results and discussion

### Structure and optical properties of (C_4_H_8_N)_2_PbCl_4_ at ambient pressure

(C_4_H_8_N)_2_PbCl_4_ is synthesized using the anti-solvent method [see the Supporting Information (SI)] and the purity is identified by powder X-ray diffraction (XRD) (Fig. S1). 1D (C_4_H_8_N)_2_PbCl_4_ is assembled with 3-pyrrolinium cations (C_4_H_8_N^+^) and [PbCl_4_]^2−^ double chains, where the organic cations are separated into 4 columns by the inorganic chains that propagate along the *b*-axis, and the inorganic octahedral unit is interlinked *via* edge-sharing (Fig. 1a and Table S1). It is worth noting that the 3-pyrrolinium cation possesses a single π electron, which endows it with potential for fluorescence emission and chemical reactivity.^25,26^ Finally, the distinctive four-column arrangement of 3-pyrrolinium cations imposes strong topochemical constraints on pressure-induced intermolecular reactions. This geometry favors intermolecular coupling within the *ac* plane over extended polymerization along the *b* direction (Fig. S2). Together with the chemically active C=C bond, this structural feature provides the basis for the unusual pressure-induced optical behaviours observed in this work. In the inorganic octahedral unit, the Pb–Cl bond lengths range from 2.61 to 3.22 Å (Table S2). The bond-length distortion (Δ*d*) and the octahedral angle variance (*σ*_oct_^2^) are calculated to be 48.35 × 10^−4^ and 19.77, respectively. These values are comparable with those of 1D Pb-based structures, such as (C_4_N_2_H_14_)PbCl_4_ (Δ*d* = 15.3 × 10^−4^, *σ*_oct_^2^ = 8.28) and (C_5_N_2_H_16_)Pb_2_Br_6_ (Δ*d* = 19.1 × 10^−4^, *σ*_oct_^2^ = 32.0).^27,28^ The significant distortion within the inorganic framework of (C_4_H_8_N)_2_PbCl_4_ is expected to facilitate the formation of STEs, thereby contributing to its broadband emission, like (C_4_N_2_H_14_)PbCl_4_ and (C_5_N_2_H_16_)Pb_2_Br_6_.^27,28^

**Fig. 1:**
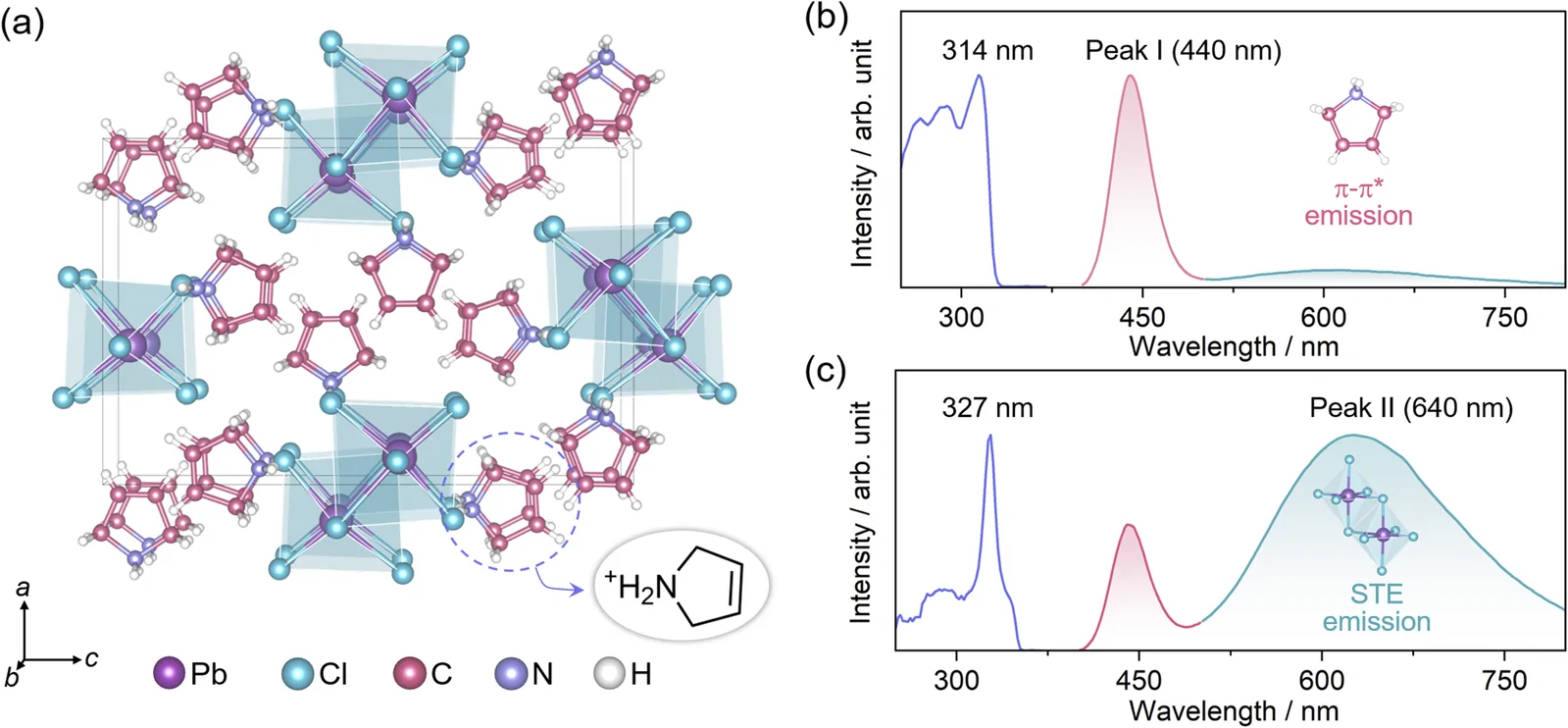
(a) Crystal structure of the 1D (C_4_H_8_N)_2_PbCl_4_. (b,c) PLE and PL emission spectra of (C_4_H_8_N)_2_PbCl_4_. The PL emission spectra are recorded under excitation at 314 nm (b) and 327 nm (c), respectively. The PLE spectrum (purple line) corresponding to Peak I is monitored at 440 nm (b), while that corresponding to Peak II is monitored at 640 nm (c).

As illustrated in Fig. 1b and c, the crystal exhibits two distinct emission peaks at 440 nm (Peak I) and 640 nm (Peak II), and the photoluminescence excitation (PLE) spectra associated with these two peaks are different, suggesting the presence of two separate luminescent centers within the crystal structure. To identify the origin of photoluminescence (PL) in (C_4_H_8_N)_2_PbCl_4_, the emission intensities of the two peaks are measured under varied excitation powers (Fig. S3). The power exponents for Peak I and Peak II are 1.35 and 1.51, respectively, implying that both peaks are non-defect-related emissions (Fig. S4).^29–31^ As shown in Fig. S5 and S6, Peak I of (C_4_H_8_N)_2_PbCl_4_ exhibits PL characteristics identical to those of its corresponding organic salt (C_4_H_7_N·HCl). This strongly supports the assignment of Peak I to the π–π* emission from 3-pyrrolinium cation. Meanwhile, Peak II exhibits a large Stokes shift of 338 nm (1.93 eV) and a large full-width at half-maximum (FWHM) of 177 nm (0.49 eV). The pronounced octahedral distortion and soft lattice in (C_4_H_8_N)_2_PbCl_4_ indicates strong electron–phonon coupling, favoring the formation of localized lattice relaxation upon photoexcitation. Combined with the observed broadband emission and large Stokes shift, these features unambiguously indicate that Peak II originates from STE emission of the inorganic component, which bears resemblance to STE emissions observed in other 1D Pb-based double edge-shared structures.^12,27,28,32^ Such dual emission, arising respectively from the organic and inorganic components, has been widely observed in HMHs containing π-electron–rich organic ligands.^33–36^

### Modification of optical properties *via* pressure engineering

As shown in Fig. 2a and S7, pressure-dependent PL spectra of the (C_4_H_8_N)_2_PbCl_4_ crystal are collected up to 12.0 GPa. Under initial compression, the intensity of Peak I gradually decreases, disappearing entirely above 2.0 GPa, while the intensity of Peak II increases significantly and reaches its maximum at 4.2 GPa. The suppressed luminescence of the 3-pyrrolinium cations (Peak I) is attributed to pressure-enhanced intermolecular π–π interactions. Compression shortens the intermolecular separation and increases orbital overlap between adjacent cations, facilitating aggregation-caused non-radiative relaxation pathways that efficiently compete with the intrinsic π–π radiative transition. This phenomenon is consistent with the established aggregation-induced quenching (ACQ) mechanism commonly observed in π-conjugated organic luminophores.^37–39^ Conversely, for the [PbCl_4_]^2−^ double chains, the pressure-enhanced STE emission—arising from increased exciton binding energy, reduced carrier scattering, and suppression of non-radiative decay channels—is fully consistent with expectations.^40^ Therefore, the luminescence of the sample varies from weak green emission (due to the combined effect of blue emission of organic components and yellow emission of inorganic components) to strong yellow emission (Fig. 2a). Above 4.2 GPa, Peak II undergoes considerable weakening in intensity (Fig. S6). Due to the continuous blueshift of Peak II, the luminescence of the sample changes from yellow to green above 7.6 GPa (Fig. 2a). It should be noted that, during decompression, Peak II still exhibits a continuous blueshift, and the luminescence of the sample varies from green to blue. Upon decompression to ambient pressure, the bimodal emissions are restored, but the energy and intensity of both emissions are irreversibly modified after pressure engineering (Fig. 2a and b). Peak I exhibits a redshift of 0.17 eV (from 441 to 469 nm) and a 9-fold increase in emission intensity, which should relate to the change in the arrangement of organic cations. However, Peak II exhibits a large blueshift of 0.40 eV (118 nm from 665 to 547 nm) and a 7-fold increase in emission intensity. As shown in Fig. 2c, under pressure engineering up to 12.0 GPa, compression induces a 0.23 eV blueshift of Peak II, while subsequent decompression produces an additional 0.17 eV blueshift. This pronounced asymmetry and unusually large irreversible modification of the STE emission is rare among HMHs under pressure engineering.^32^

**Fig. 2:**
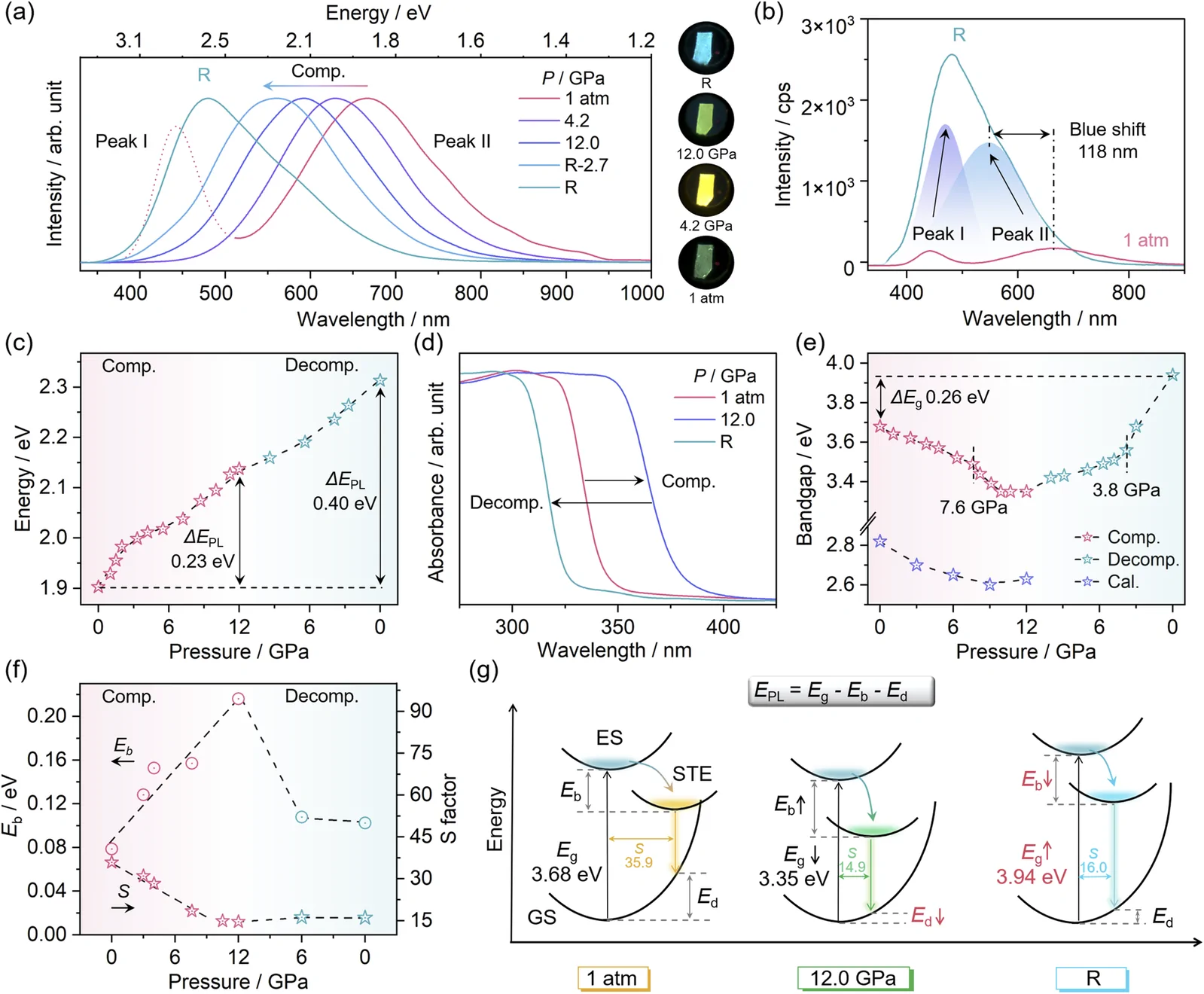
Pressure-engineered evolutions of the optical properties of (C_4_H_8_N)_2_PbCl_4_. (a) Selected pressure-dependent normalized PL spectra of (C_4_H_8_N)_2_PbCl_4_ crystal excited at 325 nm. R denotes the sample recovered to ambient pressure after decompression. Representative PL micrographs recorded during the compression–decompression cycle are shown as insets. (b) Comparison of PL spectra of (C_4_H_8_N)_2_PbCl_4_ crystal before (1 atm) and after pressure engineering (R). (c) Pressure-dependent emission energy of Peak II during a compression–decompression cycle. (d) Selected UV-Vis absorption spectra of (C_4_H_8_N)_2_PbCl_4_ crystal. (e) Bandgap evolution of (C_4_H_8_N)_2_PbCl_4_ crystal during a compression–decompression cycle and the calculation results based on the ambient pressure phase. (f) Pressure-dependent exciton binding energy and *S* factor during a compression–decompression cycle. (g) Schematic illustration of STE emission evolution in (C_4_H_8_N)_2_PbCl_4_*via* pressure engineering. Abbreviations: GS, ground state; ES, excited state; STE, self-trapped exciton. The key parameters that cause the blueshift of *E*_PL_ at different stages are highlighted in red.

The variation of the bandgap of (C_4_H_8_N)_2_PbCl_4_ during pressure engineering is explored *via in situ* ultraviolet-visible (UV-Vis) absorption spectroscopy. Fig. 2d and S8 show that the absorption edge of (C_4_H_8_N)_2_PbCl_4_ undergoes a redshift during compression and a blueshift during decompression. However, the absorption edge of the recovered sample is irreversibly blue-shifted by around 25 nm compared to the original sample, indicating an irreversible structural modification of (C_4_H_8_N)_2_PbCl_4_ following pressure treatment. As shown in Fig. 2e, upon increasing pressure up to 7.6 GPa, the bandgap of (C_4_H_8_N)_2_PbCl_4_ gradually narrows from 3.68 eV to 3.49 eV, with a relatively slow rate of change. Between 7.6 and 9.9 GPa, the bandgap decreases more sharply, after which it remains nearly constant with further compression. During decompression, the bandgap increases progressively from 11.9 GPa down to 3.8 GPa, again at a moderate rate, but rises abruptly below 3.8 GPa. The discontinuous evolution of the bandgap observed during both compression and decompression indicates pressure-induced structural modifications in (C_4_H_8_N)_2_PbCl_4_, occurring at approximately 7.6 GPa upon compression and 3.8 GPa upon decompression. This interpretation is further supported by density functional theory (DFT) calculations, which show that under ideal elastic compression the bandgap decreases gradually and subsequently exhibits only a slight increase—a trend that is inconsistent with the abrupt bandgap changes observed experimentally (Fig. 2e). The specific structural regulation mechanism requires further structural characterization for elucidation, which will be discussed in the next section. Finally, the recovered sample exhibits an increased bandgap of 3.94 eV, which is 0.26 eV larger than the initial (C_4_H_8_N)_2_PbCl_4_. To investigate the critical pressure of irreversible optical property regulation, additional decompression experiments are conducted at various pressures, including 6.0 and 7.6 GPa (Fig. S9). The results indicate that the sample could exhibit irreversible fluorescence and absorption changes at pressures above 7.6 GPa.

To evaluate the robustness of the pressure-engineered metastable state, three consecutive compression–decompression cycles are performed (Fig. S10). The recovered samples exhibit nearly identical STE emission energies after each cycle, indicating that the metastable optical state is established during the first cycle and remains stable under subsequent pressure treatments. By contrast, the gradual enhancement of PL intensity, together with the slight increase in optical bandgap, points to subtle local reconstruction of the coupled organic–inorganic lattice. This minor structural relaxation may further suppress non-radiative recombination while preserving the local environment responsible for STE formation, thereby maintaining an essentially unchanged emission energy.

In excitonic systems, the energy of STE emission (*E*_PL_) is commonly described by the relation:

1 *E*_PL_ = *E*_g_ − *E*_b_ − *E*_d_

where *E*_g_ is the bandgap, *E*_b_ is the exciton binding energy, and *E*_d_ is the lattice relaxation energy. To further understand the photophysical reasons for its asymmetric blueshift, temperature-dependent PL spectra are collected during the compression–decompression cycle (Fig. S11 and S12). *E*_b_ is determined by fitting the decay of the STE peak intensity as a function of temperature (Fig. S13),^41^ and *E*_d_ is qualitatively estimated from the Huang-Rhys factor (*S* factor), which can be derived from the linewidth broadening of STE emission at low temperatures (Fig. S11 and S12).^42–44^ As shown in Fig. 2f, during compression up to 12.0 GPa, *E*_b_ increases, whereas the *S* factor decreases. Upon decompression to ambient pressure, *E*_b_ largely recovers while the *S* factor shows only limited recovery. This behavior implies that the observed blueshift in the STE emission energy can be attributed to a sharp decrease in *E*_d_ during compression, despite the concurrent decrease in *E*_g_ and increase in *E*_b_. Conversely, during decompression, the blueshift arises primarily from a significant increase in *E*_g_ and the recovery of *E*_b_.

Fig. 2g schematically illustrates the pressure-engineered evolution of STE emission in (C_4_H_8_N)_2_PbCl_4_. At ambient pressure, the pristine sample exhibits yellow STE emission, characterized by an exciton binding energy (*E*_b_ ≈ 0.08 eV), a Huang–Rhys factor (*S* = 36), and a wide bandgap (*E*_g_ = 3.68 eV). Upon compression to 12 GPa, *S* decreases sharply to 14.6, resulting in a pronounced blueshift of the STE emission to green, despite a narrowing of *E*_g_ (3.35 eV) and an increase in *E*_b_ (0.22 eV). After decompression, the recovered *E*_b_ (0.10 eV) together with the increased *E*_g_ (3.94 eV) continue to support the STE blueshift, yielding blue emission, whereas the *S* factor (16.0(4)) shows a slight increase.

To clarify the intrinsic pressure response of the organic cations, high-pressure PL measurements on pure C_4_H_7_N·HCl that has fundamentally different crystal packing and intermolecular environments are also performed (Fig. S14 and S15). After compression to 12.0 GPa followed by decompression, the recovered sample shows reduced emission intensity but nearly unchanged emission energy. This behaviour contrasts with the pronounced optical modulation observed in (C_4_H_8_N)_2_PbCl_4_, indicating that irreversible evolution of the electronic structure and STE emission is driven by synergistic coupling between the pressure-induced organic reaction and confined inorganic framework.

### Structural evolution *via* pressure engineering

To comprehend the structural evolution of (C_4_H_8_N)_2_PbCl_4_, *in situ* XRD experiments are conducted. As shown in Fig. S16, up to 6.9 GPa, all the diffraction peaks shift to higher angles without peak emergence or disappearance, which suggests that (C_4_H_8_N)_2_PbCl_4_ maintains the orthorhombic phase from ambient pressure (*Pnma*, *a* = 12.33 Å, *b* = 6.08 Å, *c* = 18.94 Å) to 6.9 GPa. Above 9.0 GPa, a new diffraction peak appears at 4.1° (8.65 Å) and its intensity increases significantly with increasing pressure (Fig. 3a). Given that the newly formed diffraction peak is located close to the (002) reflection, it probably originates from structural changes along the *c*-axis in the new phase. The *Pnma* phase persists up to 13.4 GPa, as indicated by the lattice parameters obtained from Rietveld refinement, and the new diffraction peak originates from the appearance of a new high-pressure (HP) phase (Fig. S17 and 3a). The *P*–*V* relationship of (C_4_H_8_N)_2_PbCl_4_ is then fitted using a 3rd-order Birch–Murnaghan equation of state with *V*_0_ = 1449(19) Å^3^, *B*_0_ = 20.2 (1.2) GPa, and *B*_1_ = 4 (fixed) (Fig. S18).^45^ Upon further compression to 16.6 GPa, the (002) diffraction peak of the *Pnma* phase disappears completely and the diffraction peak at 4.1° originating from the (101) peak also experiences an abnormal shift to lower angles (Fig. 3a). This suggests that the *Pnma* phase may have fully transformed into the new HP phase featuring a distinct periodicity of the inorganic chains, while the observed anomalous expansion is most likely associated with structural adjustments in the organic framework, just like other π-bond-initiated polymerization processes in hybrid materials.^7–9^ Due to data quality limitations, the HP phase could not be resolved at this moment. During decompression, the HP phase does not return to the original *Pnma* phase but transforms into a metastable phase with disordered [PbCl_4_]^2−^ chains (Fig. 3b). These results suggest that pressure engineering preserves only the (101)-plane ordering of the original *Pnma* phase, which corresponds to the interchain periodicity (10.47 Å) of the [PbCl_4_]^2−^ chains, while the intrachain structural coherence is lost (Fig. 3c).

**Fig. 3:**
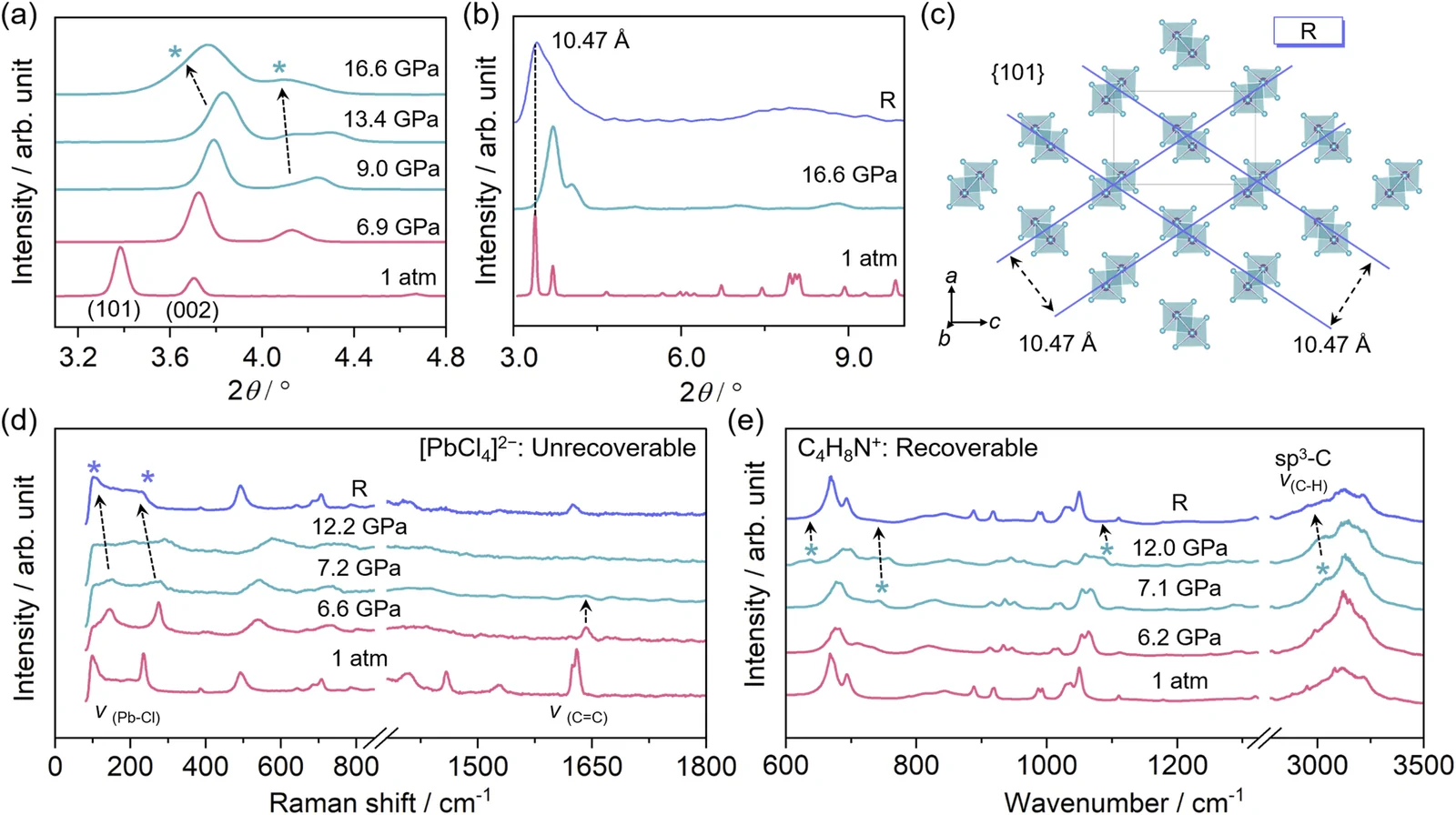
(a) Selected *in situ* XRD patterns in the region where the diffraction peaks change dramatically. (b) The comparison of *in situ* XRD patterns of (C_4_H_8_N)_2_PbCl_4_ at 1 atm, 16.6 GPa, and the recovered sample (R). (c) Crystal structure viewed along the *b* axis, highlighting that the {101} plane family mainly cuts through the [PbCl_4_]^2−^ double chains. C_4_H_8_N^+^ cations are omitted for clarity in crystal structure. Selected *in situ* Raman (d) and IR (e) spectra of (C_4_H_8_N)_2_PbCl_4_ under compression. R represents the recovered sample at ambient pressure. The spectral features associated with the structural evolution are marked with asterisks.

To gain deeper insight into the structural evolution under pressure, *in situ* Raman and Infrared (IR) spectra are collected (Fig. 3d–e, and S19–S20). As shown in Fig. 3d, the two vibration modes in the low-frequency range (100–300 cm^−1^) are attributed to the vibrations of the inorganic [PbCl_4_]^2−^ chains due to their large reduced mass. Above 7.2 GPa, their intensity diminishes and the peaks broaden, reflecting the onset of disorder in the inorganic polyhedra. Simultaneously, the *ν*_(C=C)_ stretching mode of the C_4_H_8_N^+^ cations disappears, suggesting disruption of the π-bonds within the molecular framework. Upon decompression from 12.2 GPa, the characteristic Raman features of the sample are not fully recovered: the inorganic-related vibrations remain broadened, while the *ν*_(C=C)_ mode reappears with significantly reduced intensity. The intensity loss and spectral broadening of the Raman modes can be attributed to the intrinsic sensitivity of Raman scattering to long-range structural order,^46^ confirming the partial loss of structural coherence after pressure release. In the *in situ* IR spectra (Fig. 3e), several new absorption bands emerge above 7.1 GPa (highlighted by green asterisks), which are attributed to newly activated vibrations of the organic cations. Their appearance implies a pressure-induced modification of the C_4_H_8_N^+^ molecular skeleton. More importantly, the enhanced *ν*_(C–H)_ stretching of sp^3^-hybridized carbons in the IR spectra, together with the disappearance of *ν*_(C=C)_ in the Raman spectra, strongly suggests that the structural transformation is driven by polymerization across the C=C bonds of the organic cations. The onset pressure of this process (7.1–7.2 GPa) is in excellent agreement with the critical pressure (∼7.6 GPa) at which irreversible PL and absorption changes occur, confirming that the optical transformation is reaction-driven rather than purely structural. After decompression from 12.0 GPa, the IR modes of C_4_H_8_N^+^ largely recover, indicating that the pressure-induced chemical reactions are reversible under confinement. Such reversibility of carbon–carbon polymerization is exceptionally rare and likely results from the constrained environment of the inorganic framework, which suppresses the stabilization of extended polymer chains. Further discussion of this confinement-assisted reversibility is provided in Fig. S21 and 22. Nevertheless, the recovery of the molecular skeleton does not imply complete restoration of the crystal structure. Instead, the coupled organic–inorganic framework retains subtle local reconstruction after decompression, as evidenced by the incomplete recovery of the Raman features and the persistent optical changes.

Taken together, these observations indicate that the recovered sample should be regarded as a kinetically trapped metastable state rather than a new thermodynamic phase. The pressure-induced local reconstruction arises from the cooperative coupling between the confined 3-pyrrolinium cations and the flexible Pb–Cl framework, producing a metastable organic–inorganic configuration that is preserved by kinetic barriers after decompression. Consequently, the pressure-induced optical properties can persist under ambient conditions. In contrast, dissolution would disrupt the long-range crystal packing and eliminate the cooperative organic–inorganic structural arrangement, thereby erasing the pressure-induced structural memory. Subsequent recrystallization would proceed under thermodynamic control, regenerating the energetically favored pristine structure rather than the pressure-induced metastable state.

Ultimately, pressure engineering enables persistent optical modification in (C_4_H_8_N)_2_PbCl_4_ through irreversible reconstruction of the coupled organic–inorganic lattice. Although the molecular skeleton of the 3-pyrrolinium cations is largely restored after decompression, the recovered structure remains metastable with disordered [PbCl_4_]^2−^ chains, as evidenced by persistent changes in the XRD and Raman spectra. This reconstruction permanently increases both *E*_b_ and *E*_g_, indicating an irreversible modification of the electronic structure. As a result, the reconstructed organic sublattice suppresses non-radiative relaxation, whereas the reconstructed inorganic framework promotes exciton localization and radiative recombination. Together, these effects give rise to the enhanced dual emission and the unusual continuous blueshift of the STE emission.

## Conclusions

In summary, the *in situ* XRD and spectroscopic measurements reveal two distinct structural transitions during compression–decompression cycling, both driven by pressure-induced chemical reactions of the C_4_H_8_N^+^ cations. Owing to the intrinsically soft and deformable lattice of HMHs, perturbations originating from reversible reactions within the organic sublattice are efficiently transmitted to the inorganic framework, promoting a progressive loss of long-range structural order. As a result, the crystalline *Pnma* phase is ultimately converted into a metastable phase featuring disordered [PbCl_4_]^2−^ chains, thereby escaping the conventional reversible thermodynamic response expected under pressure. More broadly, our results highlight how the combination of lattice softness and pressure-activated organic chemistry enables HMHs to access non-equilibrium structural states beyond reversible thermodynamic cycles, offering a new paradigm for pressure-enabled, irreversible optoelectronic tuning. In summary, we uncover an asymmetric and irreversible STE response in the 1D HMH (C_4_H_8_N)_2_PbCl_4_ under pressure cycling. The STE emission undergoes a continuous blueshift throughout compression and decompression, yielding a permanent energy increase of 0.40 eV governed by distinct pathway-dependent mechanisms: a reduction in lattice distortion energy during compression, and a persistently widened bandgap together with recovered exciton binding energy upon decompression. This behavior originates from pressure-driven structural mutations of the inorganic [PbCl_4_]^2−^ chains cooperatively coupled with reactions of the confined organic cations. These findings establish pressure engineering as a powerful route beyond elastic lattice compression for accessing irreversible excitonic modulation in hybrid materials.

## Experimental section

### Synthetic procedures of (C_4_H_8_N)_2_PbCl_4_

Materials: Lead chloride (PbCl_2_, 99%, Bidepharm), 2,5-dihydro-1*H*-pyrrole hydrochloride (C_4_H_7_N·HCl, 97%, Bidepharm), and *N*,*N*-dimethylformamide (DMF, >99.5%, Yonghua), ethyl ether (EE, >99.7%, Yonghua). All chemical reagents in this work were commercially purchased without further purification.

(C_4_H_8_N)_2_PbCl_4_ crystals were synthesized *via* the anti-solvent diffusion method.^47^ The precursor solution was prepared by dissolving PbCl_2_ (0.1 mmol, 27.8 mg) and 2,5-dihydro-1*H*-pyrrole hydrochloride (C_4_H_7_N·HCl, 0.2 mmol, 21.0 mg) with 2 mL DMF in 4 mL glass vial by sonication for 5 min to obtain a homogeneous solution. The vial containing the precursor solution was then placed into a 20 mL glass vial containing 5 mL EE anti-solvent. After sealing the 20 mL glass vial overnight, the colorless plate crystals were synthesized. The as-prepared crystals were then filtered and dried at 40 °C for 2 h. The as-synthesized (C_4_H_8_N)_2_PbCl_4_ sample remains structurally stable after 6 months exposure to ambient conditions, indicating good environmental stability.

### Diamond anvil cell (DAC) loading

For the *in situ* high-pressure Raman, IR, powder XRD, PL and UV-Vis absorption experiments, a symmetrical-style DAC fitted with diamonds polished to a culet diameter of *d*_culet_ = 400 µm was used to apply pressure. The T-301 stainless steel gaskets were pre-indented to ∼50 µm, and holes with a diameter of *d* = 200 µm were drilled at the center of the indentation to serve as the sample chambers. Silicon oil (Aldrich, ∼150 mPa·s) was used as a pressure-transmitting medium (PTM) in PL and UV-Vis absorption experiments. In the other experiments, powder samples were filled into sample chambers without using PTM. In IR experiments, to avoid too strong infrared absorption, potassium bromide (KBr) powder was used for sample thinning. Type-II diamond anvils were used for the IR, PL and UV-Vis absorption measurements. The pressure was calibrated using the ruby fluorescence in the experiments.^48^

### Characterization

Single crystal XRD data were collected under ambient pressure at 100 K on a Bruker D8 Venture X-ray single crystal diffractometer using monochromatic Mo K*α* radiation (*λ* = 0.71073 Å). Data reduction, scaling, and absorption corrections were performed by using SAINT (Bruker, V8.38A, 2013). The data were solved with the ShelXT (Sheldrick, 2015) structure solution program by using the intrinsic phasing solution method and Olex2 was used as the graphical interface.^49^ The models were refined with version 2016/6 of ShelXL (Sheldrick, 2008) using Least Squares minimization method.^50^ Lab powder XRD patterns were recorded on an X-ray diffractometer (Rigaku Smart Lab) with Cu K*α* radiation (*λ* = 0.15418 nm) at a voltage of 45 kV and a current of 200 mA. The synchrotron powder XRD data were collected at beamline 15U1 of the Shanghai Synchrotron Radiation Facility (SSRF). The incident X-ray beam was monochromatized to a wavelength of 0.6199 Å, and the CeO_2_ was applied to calibrate the geometry parameters. Dioptas software was used to reduce the collected data.^51^ Rietveld refinement was performed using Jana 2006 software.^52^ Off-situ PL and PLE spectra were performed using the Edinburgh Instrument FLS-1000 spectrometer equipped with the excitation source of xenon lamp (450 W) at room temperature (RT). *In situ* high-pressure PL spectra were measured by the NOVA spectrometer with the laser excitation at 325 nm. The PL micrographs were captured with the same exposure time for the same run of the high-pressure experiment, using the CCD camera equipped on the microscope. For high-pressure and low-temperature experiments, the DAC was placed in a cryogenic vacuum chamber and clamped to the cold-finger of a liquid nitrogen flow cryostat (ST-500 from Janis). For UV-Vis absorption measurement, the fiber spectrometer of Ocean Optics QE6500 and a deuterium-halogen light source were applied to detect the absorption spectra. Raman spectra are collected on a HORIBA iHR550 spectrometer using a laser with wavelength at 532 nm. IR spectra are measured on a Bruker VERTEX 70v IR spectrometer with a HYPERION 2000 microscope. A Globar is used as a conventional source. The spectra are collected in transmission mode in the range of 600–4000 cm^−1^ with a resolution of 4 cm^−1^ through a 20 × 20 µm^2^ aperture. The spectrum of an empty DAC at ambient pressure in the same aperture region is used as the background.

### DFT calculations

DFT calculations were performed using the plane-wave pseudopotential method (PAW) as implemented in the VASP (Vienna Ab Initio Simulation Package).^53,54^ The gradient approximation (GGA) formulated by Perdew, Burke, and Ernzerhof was used as the exchange-correlation functional for structural optimization.^55^ The optimized kinetic energy cutoff of 500 eV and the *k*-points mesh with a grid spacing of less than 2π × 0.048 Å^−1^ were used. The atomic structures were fully relaxed until the total force on each atom was <0.03 eV Å^−1^. The DFT-D3 method was adopted to describe the long-range van der Waals interaction between the organic cations and the inorganic frameworks.^56,57^ The high pressure configurations were optimized starting from the ambient-pressure structure under applied pressure.

## Author contributions

P. Z.: conceptualization, investigation, funding acquisition, writing – original draft, methodology, data curation, formal analysis; X. X.: investigation, writing – original draft, methodology, data curation, formal analysis; Y. L: data curation, methodology; J. H.: data curation; Y. W.: data curation; Z. Q.: conceptualization, investigation, funding acquisition, writing – review and editing, project administration, formal analysis, supervision.

## Conflicts of interest

There are no conflicts to declare.

## Supplementary Material

SC-017-D6SC04557J-s001

SC-017-D6SC04557J-s002

## Data Availability

CCDC 2337630 ((C_4_H_8_N)_2_PbCl_4_) contains the supplementary crystallographic data for this paper.^58^ Supplementary information (SI): data analyses, supplementary figures and tables. See DOI: https://doi.org/10.1039/d6sc04557j.
