# Supplementary material for: Continuous blueshift of self-trapped exciton emission upon compression and decompression in one-dimensional (C4H8N)2PbCl4
Source: Chem Sci. 2026 Aug 15;17(40):19180–8. doi: 10.1039/d6sc04557j (PMC13508816; doi:10.1039/d6sc04557j)
Supplement: Supplementary file 1 [file SC-017-D6SC04557J-s001.pdf]

Supplementary Information (SI) for Chemical Science.
This journal is © The Royal Society of Chemistry 2026

Supporting Information

Continuous Blueshift of Self-Trapped Exciton Emission upon Compression and Decompression in One-Dimensional $(C_4H_8N)_2PbCl_4$

*Peijie Zhang,[#] Xiaofan Xu,[#] Yawen Li, Jiang Han, Yiyang Wang, Zewei Quan,**

Data analyses:

The direct bandgap value was analyzed based on the Tauc plot as the equation:

$$(Ahv)^2 \propto hv$$

where A denotes the absorbance, hv represents the incident photon energy.

Exciton binding energy (E_b) of $(C_4H_8N)_2PbCl_4$ is evaluated by fitting the following equation:

$$I = I_0 / \left[1 + A\, exp(-\frac{E_{b,STE}}{k_B T}) \right]$$

where I is integrated PL intensity, T is temperature, and k_B is the Boltzmann constant.

The Huang–Rhys factor (S) of $(C_4H_8N)_2PbCl_4$ was evaluated by fitting the temperature-dependent full width at half maximum (FWHM) of the STE emission according to the following equation:

$$\mathrm{FWHM} = 2.36\sqrt{S}\hbar\omega \sqrt{coth\left(\frac{\hbar\omega}{2k_B T}\right)}$$

where $\hbar$ is the reduced Planck constant, ω is the phonon frequency, T is temperature, and k_B represents the Boltzmann constant.

Lattice parameters were obtained from the Rietveld refinements of XRD data. The pressure-volume (P-V) data were fitted using the third-order Birch-Murnaghan (B-M) equation of state：

$$P = \frac{3B_0}{2}\left[\left(\frac{V_0}{V}\right)^{\frac{7}{3}} - \left(\frac{V_0}{V}\right)^{\frac{5}{3}}\right] \times \left\{1 + \frac{3}{4}(B_0^{'} - 4)\left[\left(\frac{V_0}{V}\right)^{\frac{2}{3}} - 1\right]\right\}$$

where V_0 is the zero-pressure volume, B_0 is the bulk modulus, and $B_0^{'}$ is the first pressure derivative of B_0 (fixed to 4).

Supplementary Figures

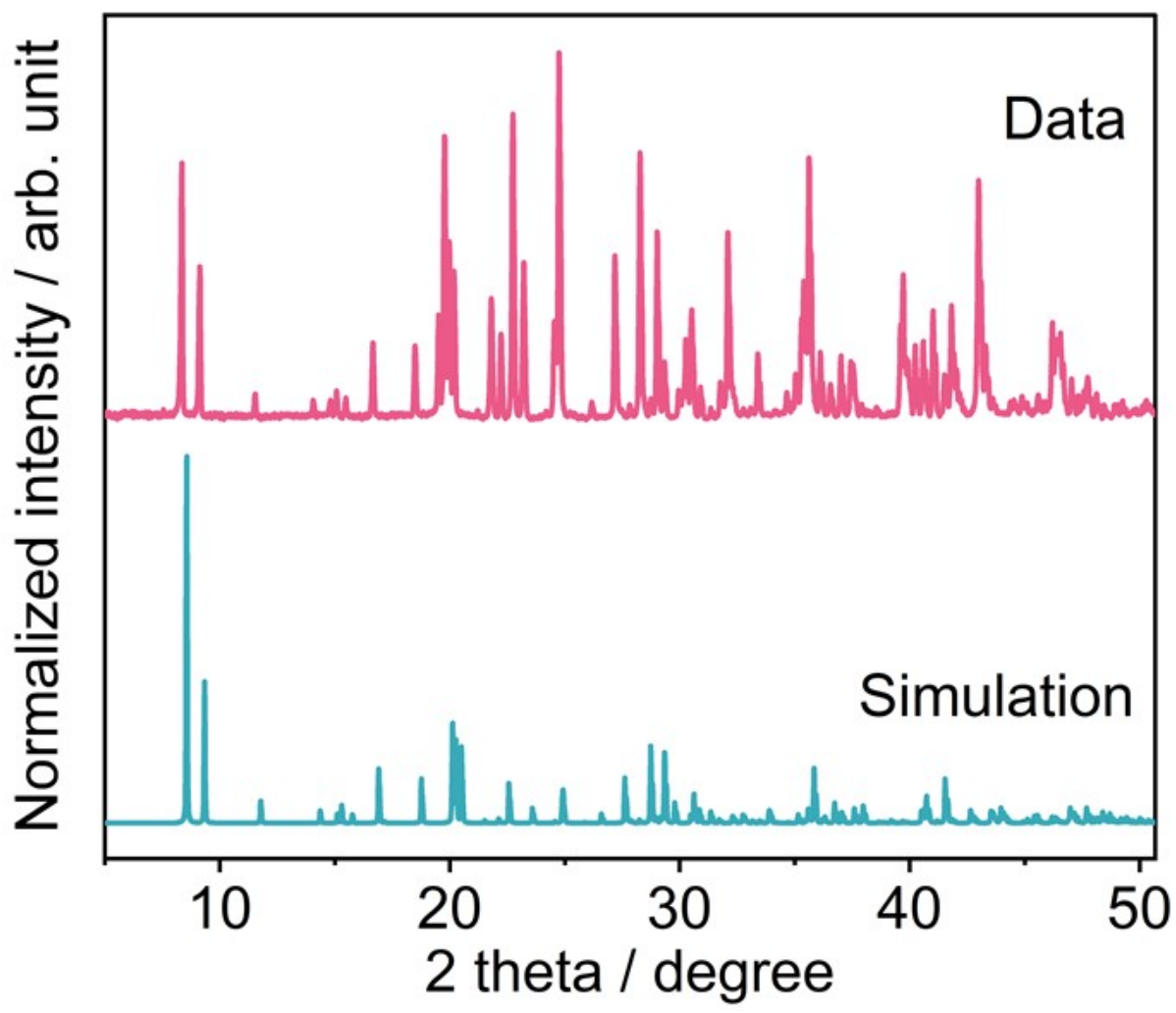

Figure S1. Powder XRD pattern and simulated pattern for $(C_4H_8N)_2PbCl_4$.

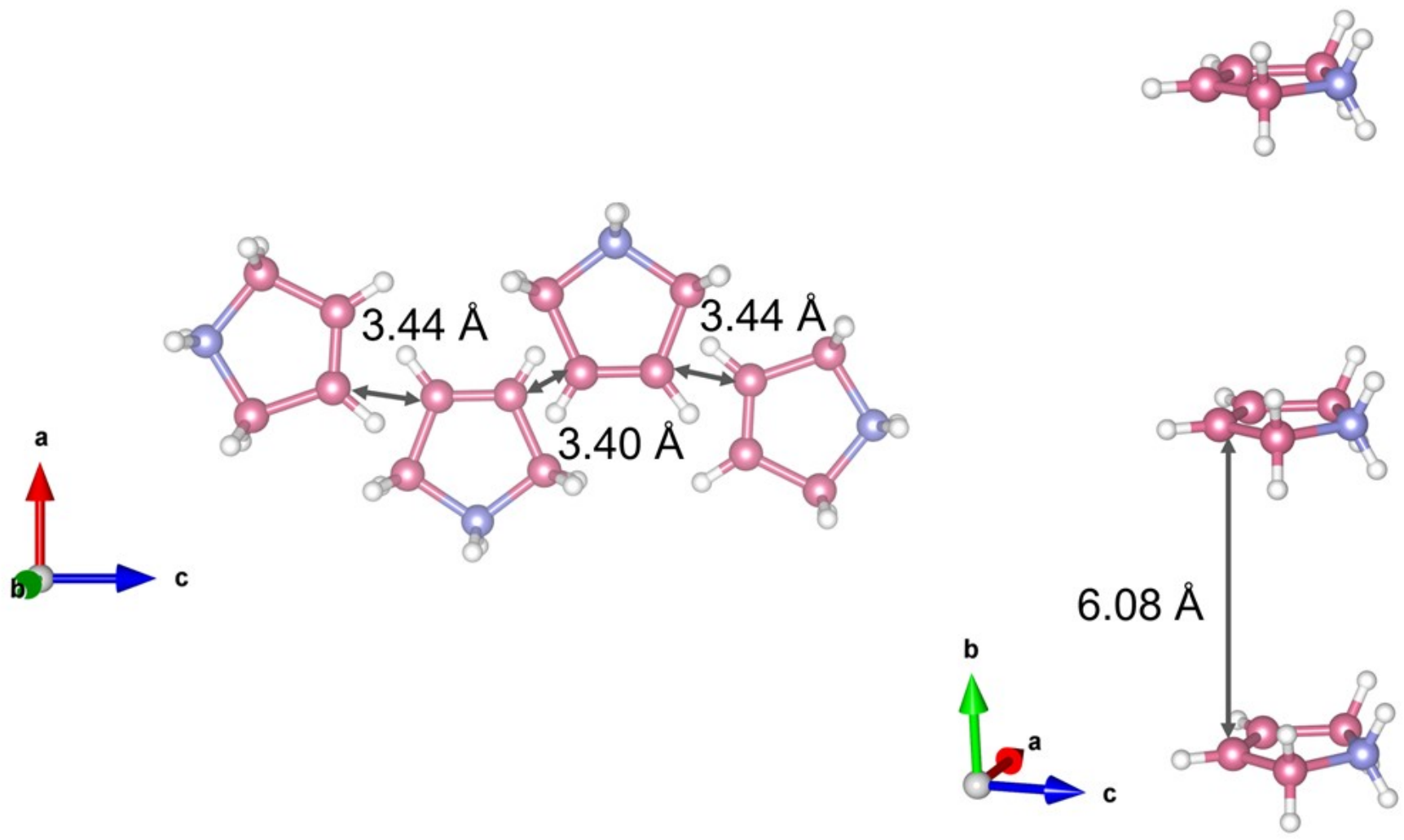

Figure S2. Nearest-neighbor distances between sp^2 carbon atoms along different crystallographic directions. Pink, light blue, cyan, and white spheres denote C, N, Cl, and H atoms, respectively. The potential reaction distance in the *ac* plane is substantially shorter than that along the *b* axis.

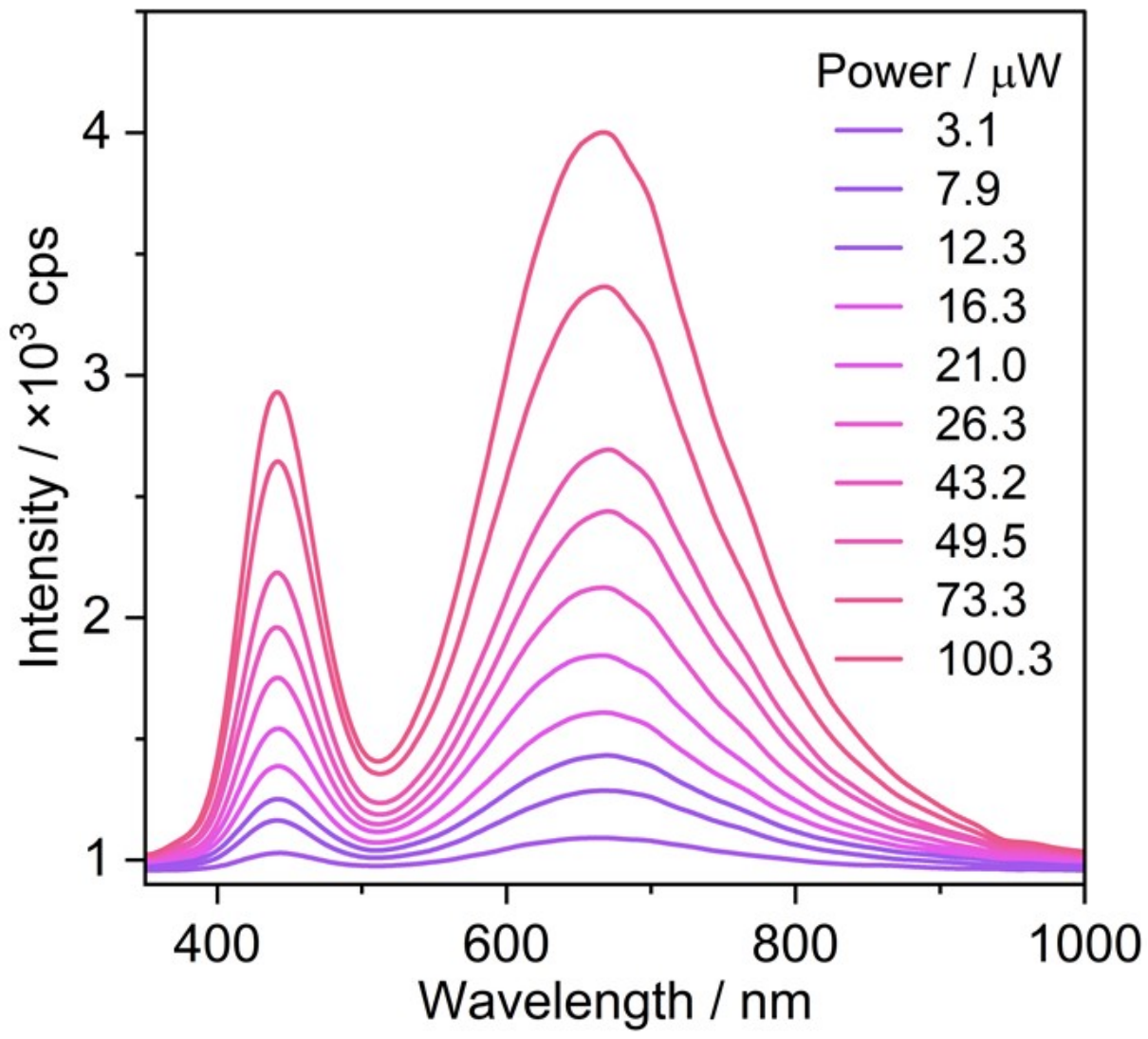

Figure S3. The power-dependent PL spectra of $(C_4H_8N)_2PbCl_4$ at ambient conditions. The PL signals are detected under excitation at 325 nm.

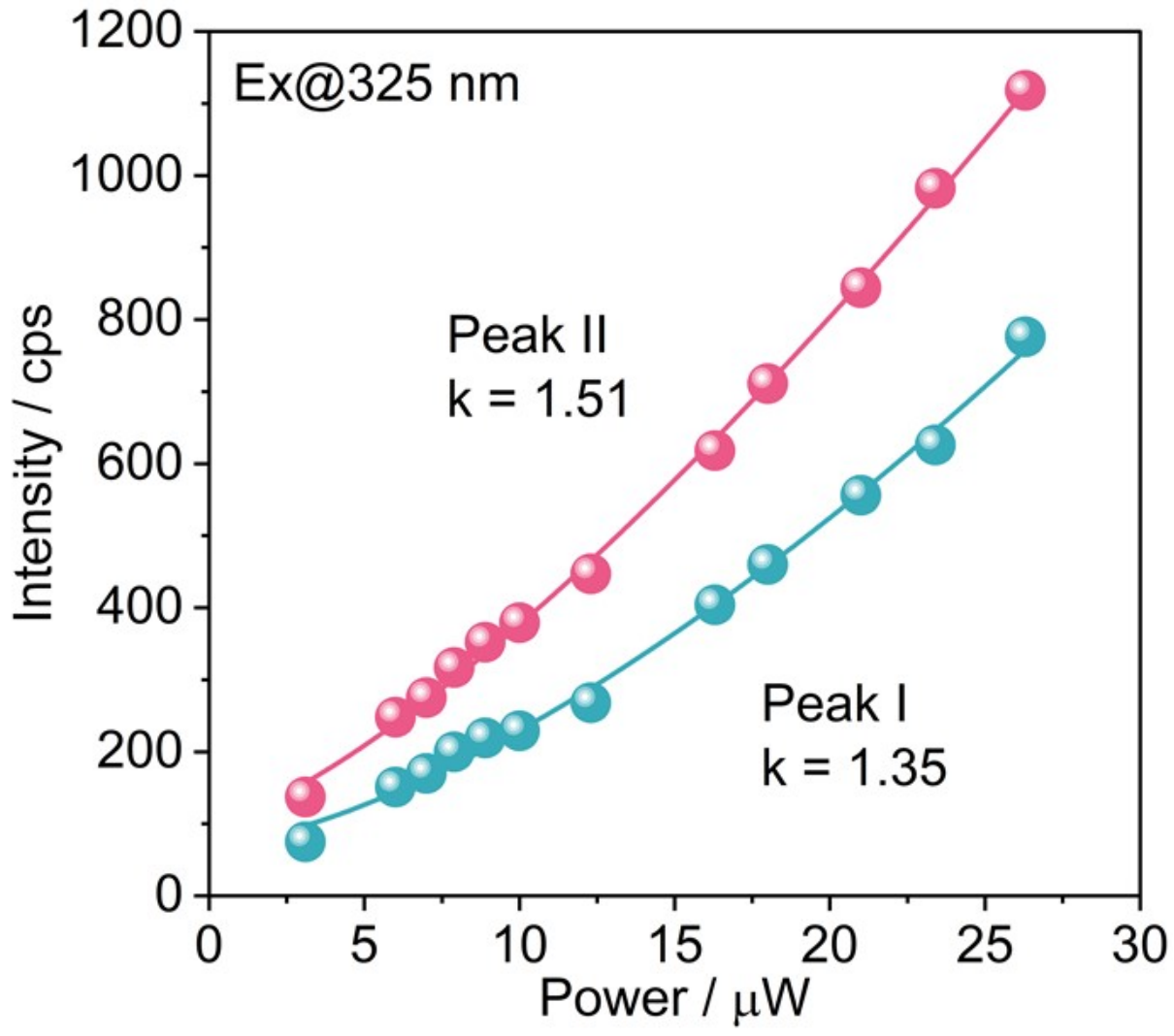

Figure S4. The corresponding PL intensities of Peak I and Peak II with the excitation power. The solid lines represent the data fittings with the power-law equation $I \propto L^k$.

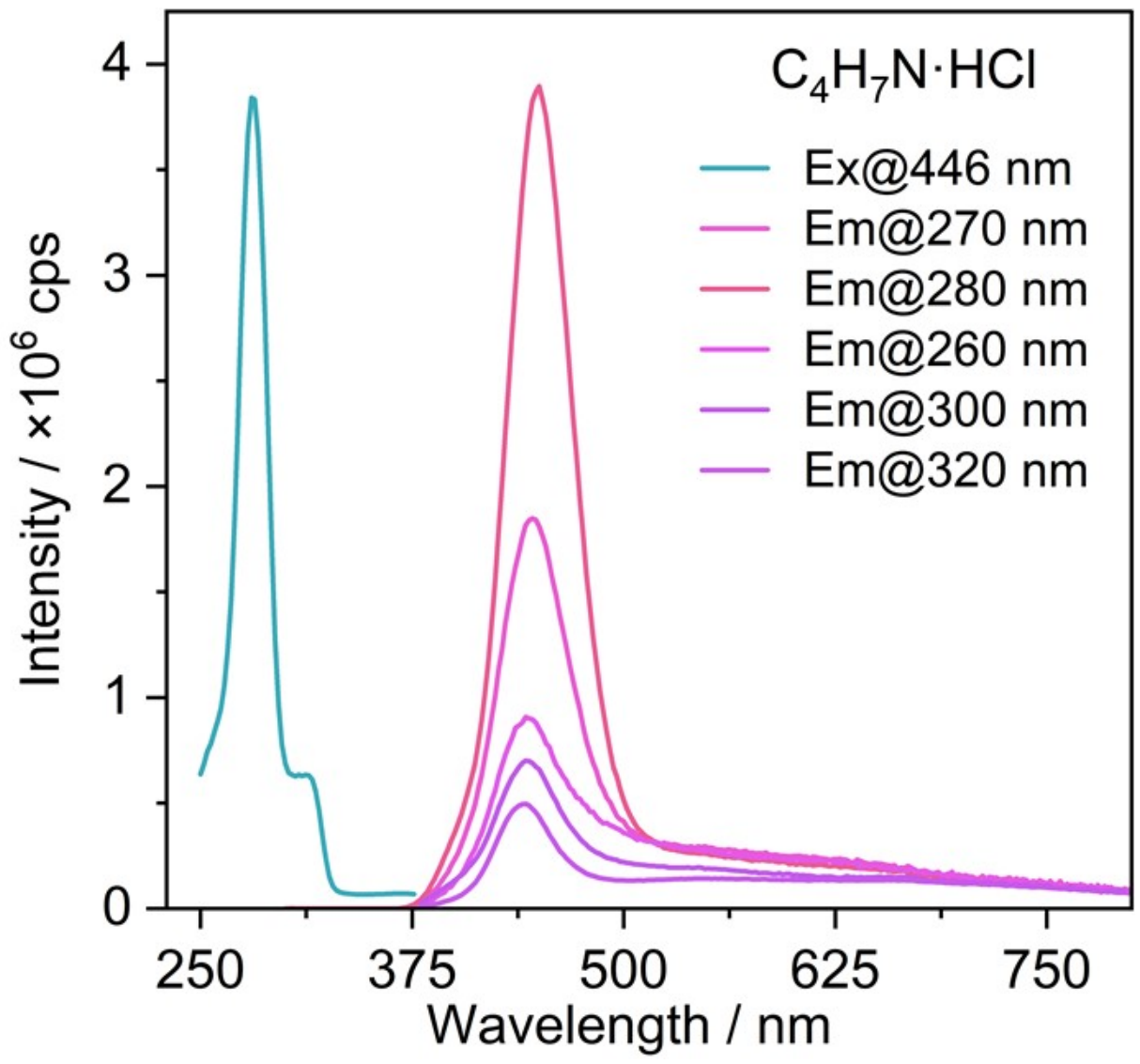

Figure S5. The excitation and emission spectra of $C_4H_7N{\cdot}HCl$ under different excitation wavelengths at 80 K.

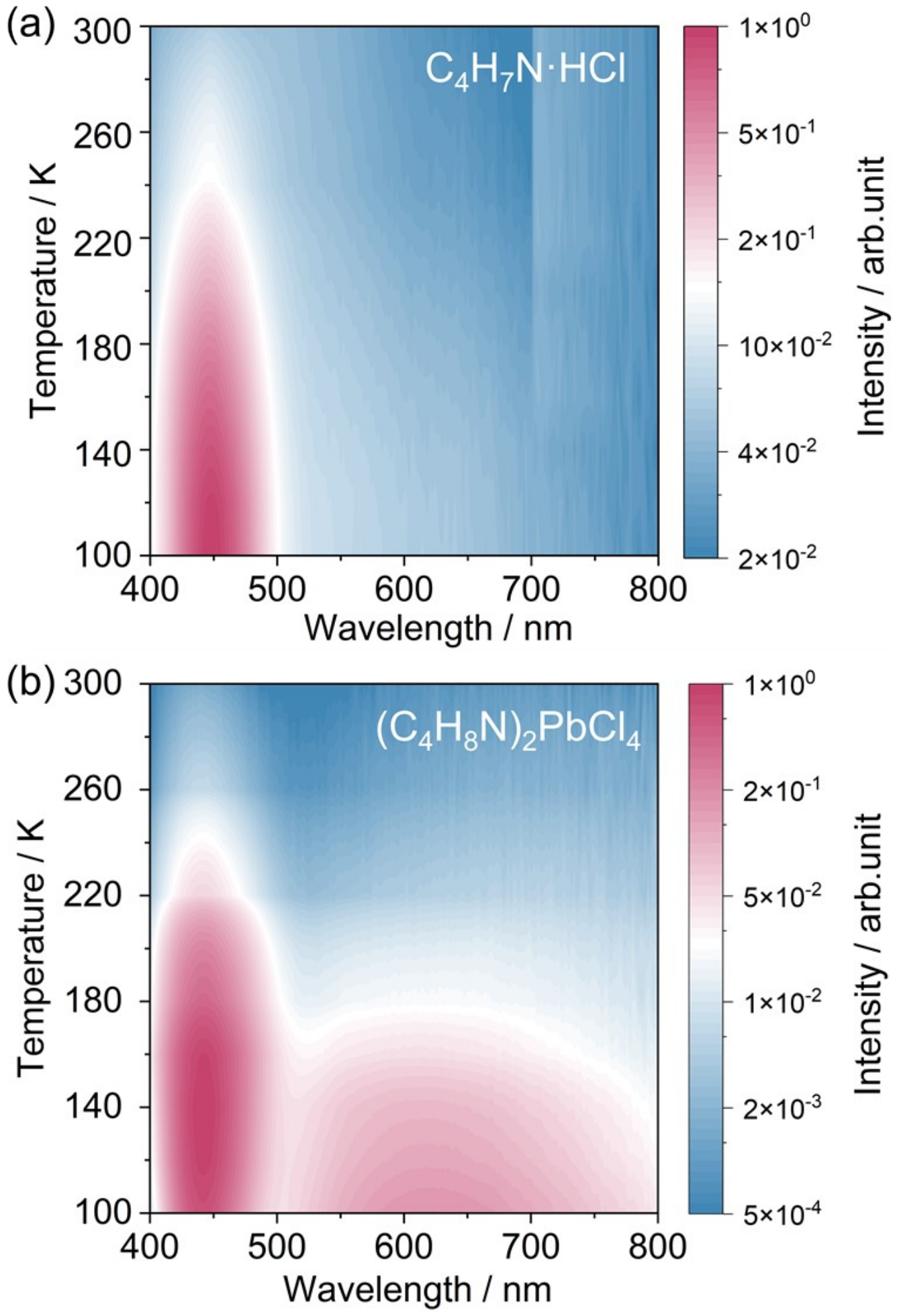

Figure S6. The temperature-dependent PL spectra of (a) $C_4H_7N{\cdot}HCl$ excited at 280 nm and (b) $(C_4H_8N)_2PbCl_4$ excited at 325 nm.

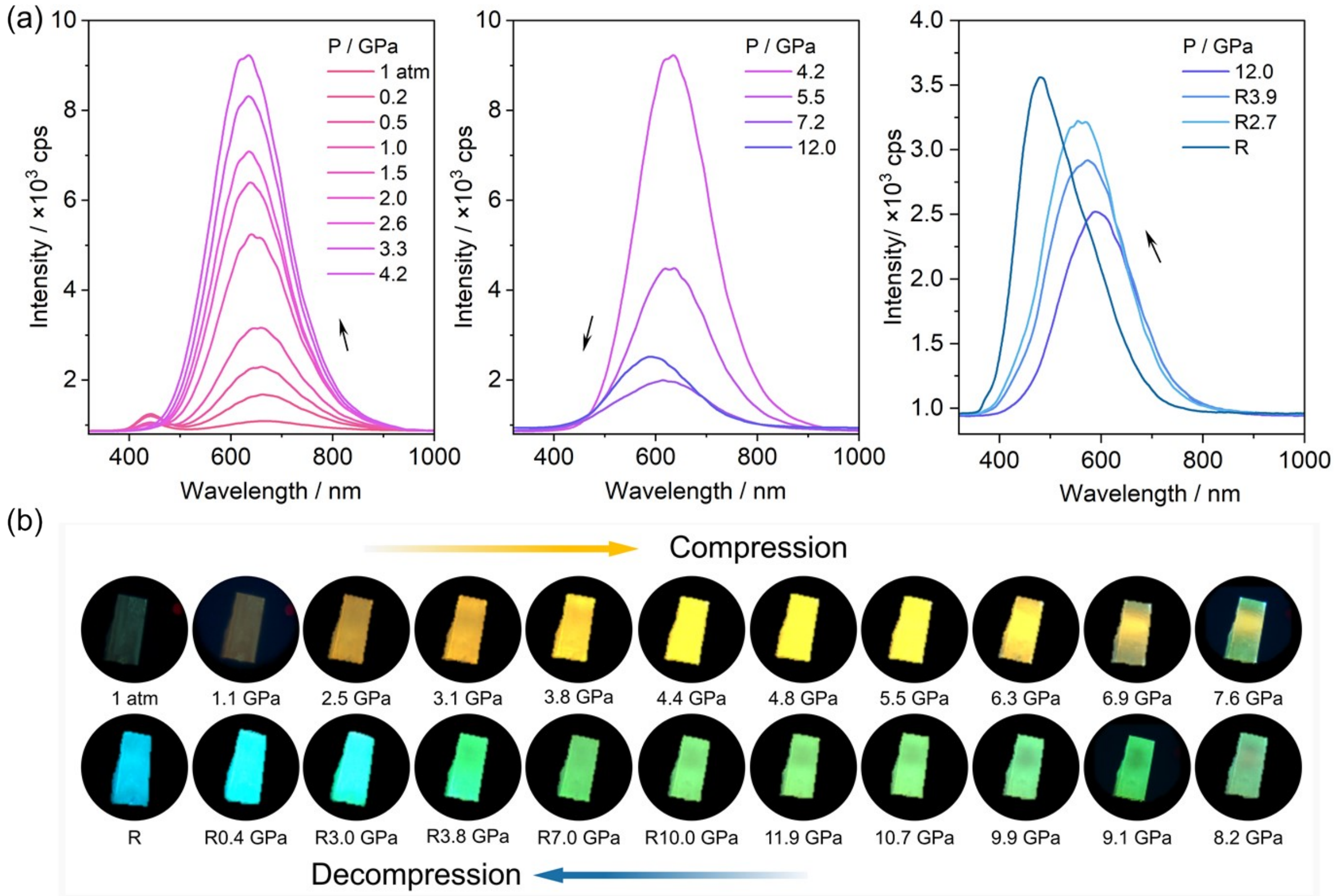

Figure S7. (a) Pressure-dependent PL spectra of $(C_4H_8N)_2PbCl_4$ crystal excited at 325 nm. (b) PL photographs during compression and decompression. "R" preceding a pressure value denotes decompression, while "R" alone denotes the fully decompressed state at ambient pressure.

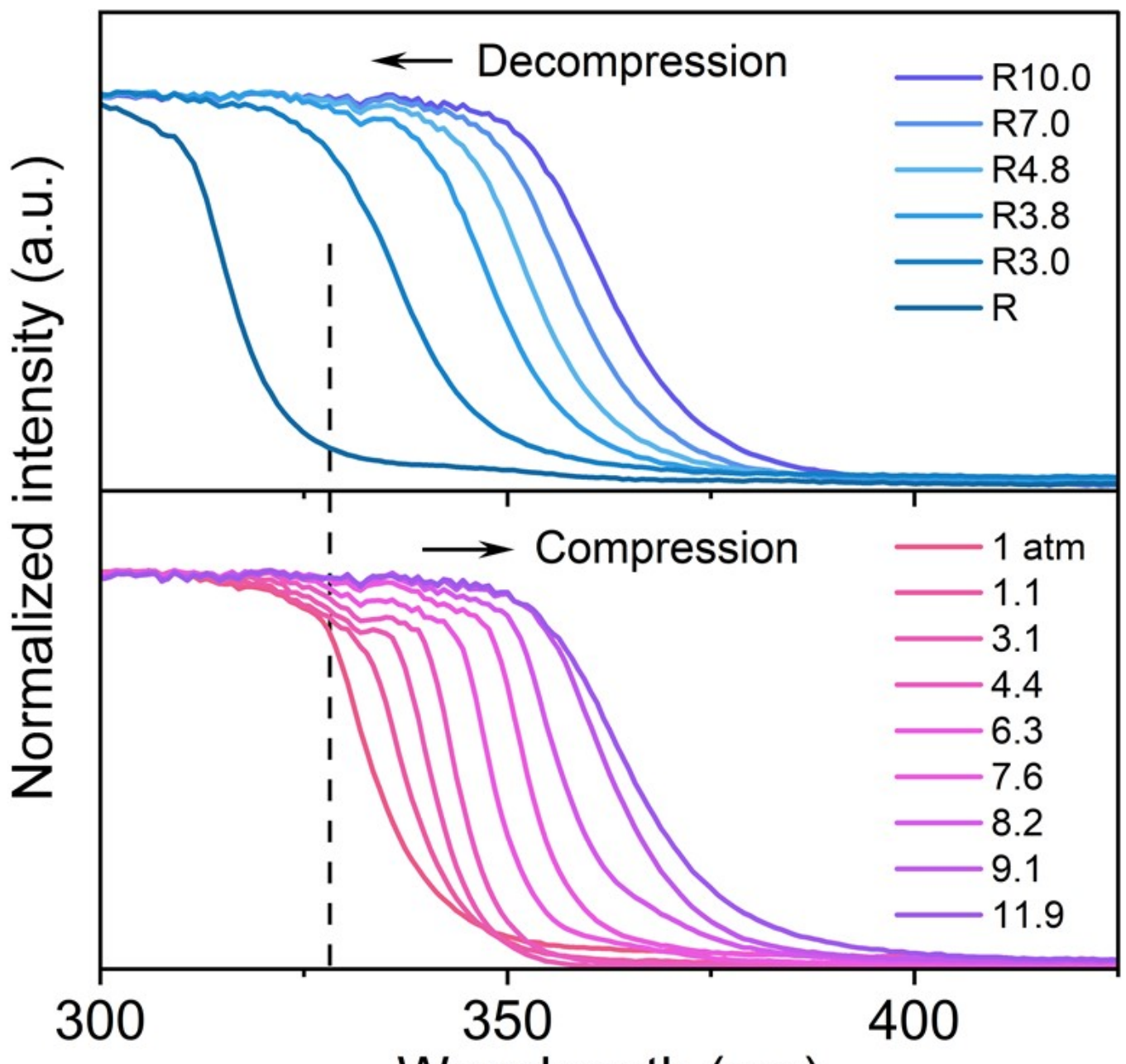

Figure S8. In situ high-pressure UV-Vis absorption spectra of $(C_4H_8N)_2PbCl_4$.

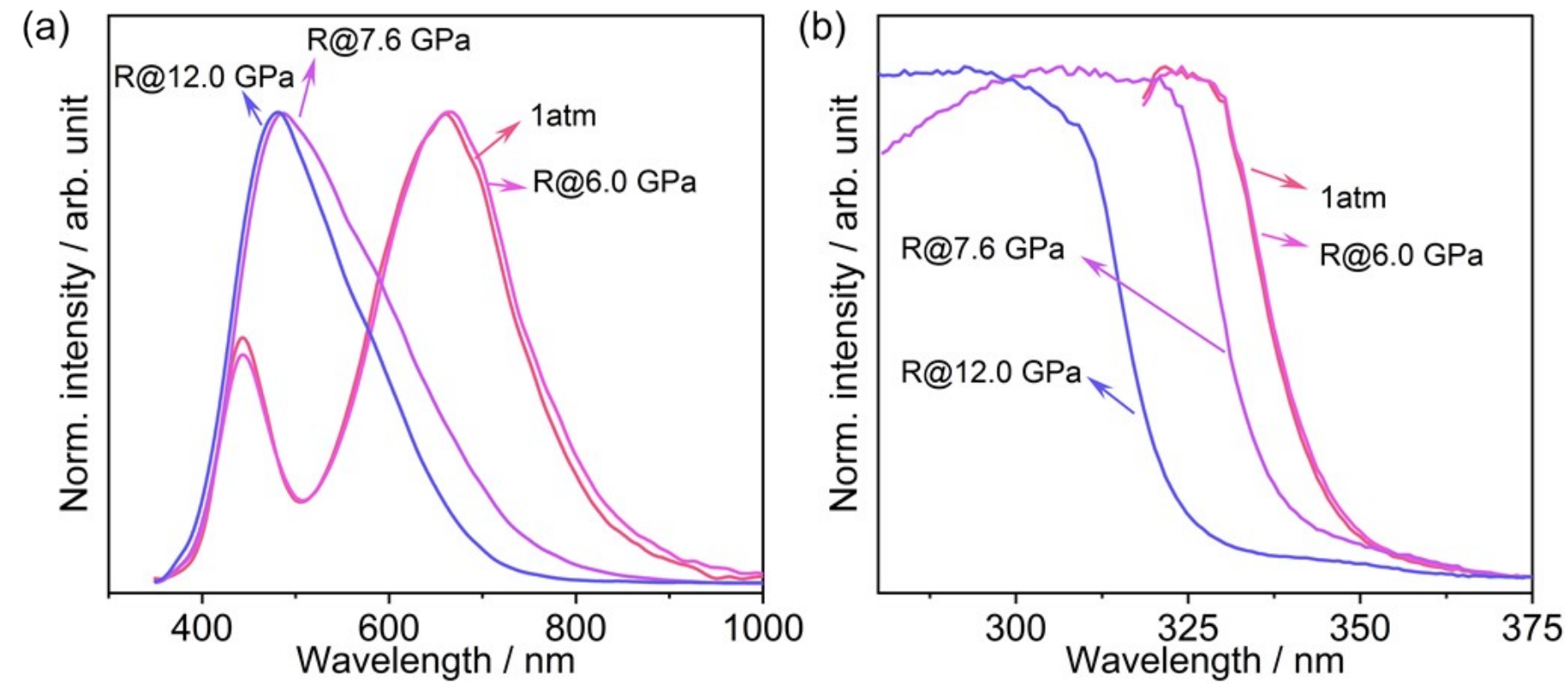

Figure S9. (a) PL spectra and (b) UV-Vis absorption spectra of $(C_4H_8N)_2PbCl_4$ crystal at 1 atm and after decompression from different pressures.

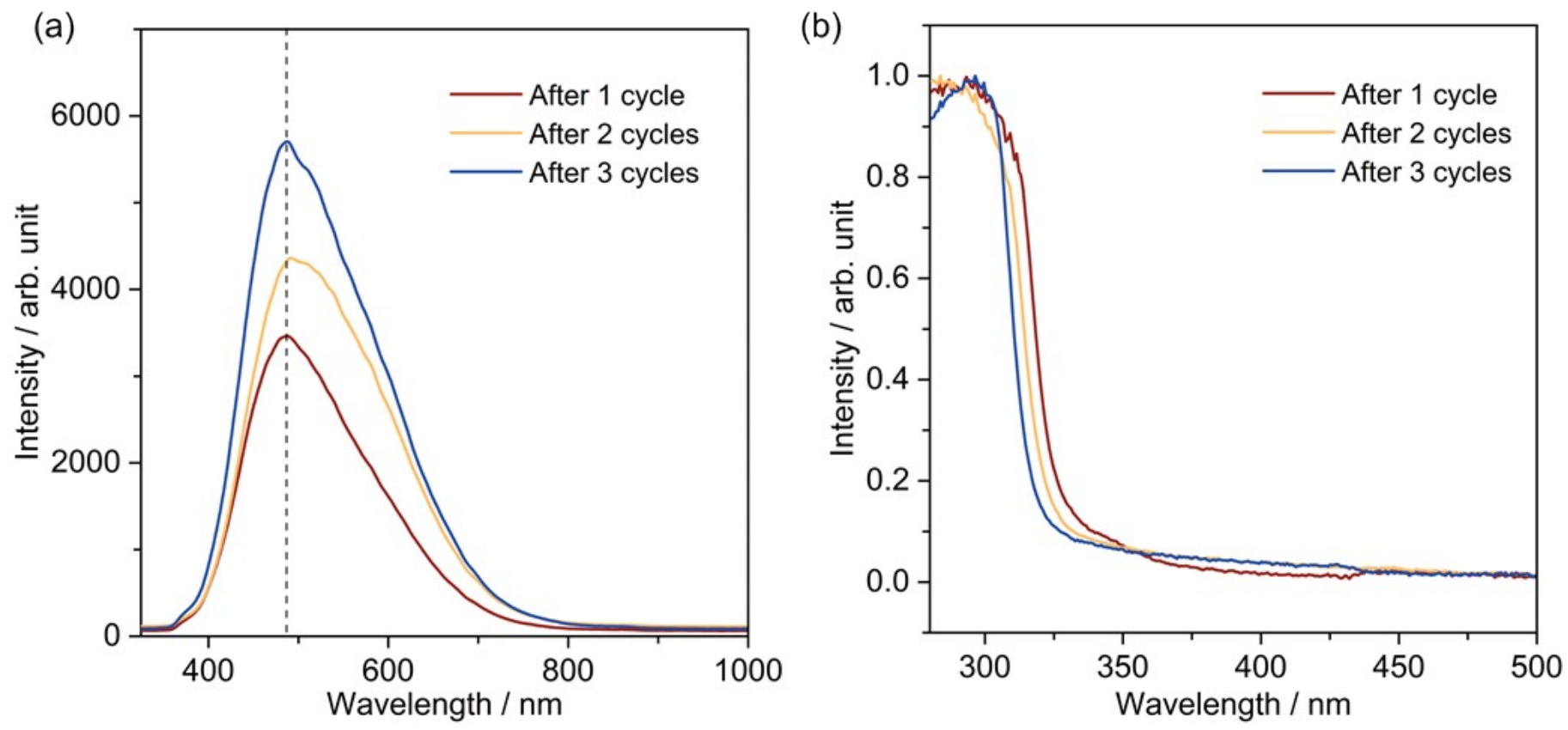

Figure S10. (a) PL spectra and (b) UV-Vis absorption spectra of $(C_4H_8N)_2PbCl_4$ crystal after three consecutive compression–decompression cycles up to 12 GPa.

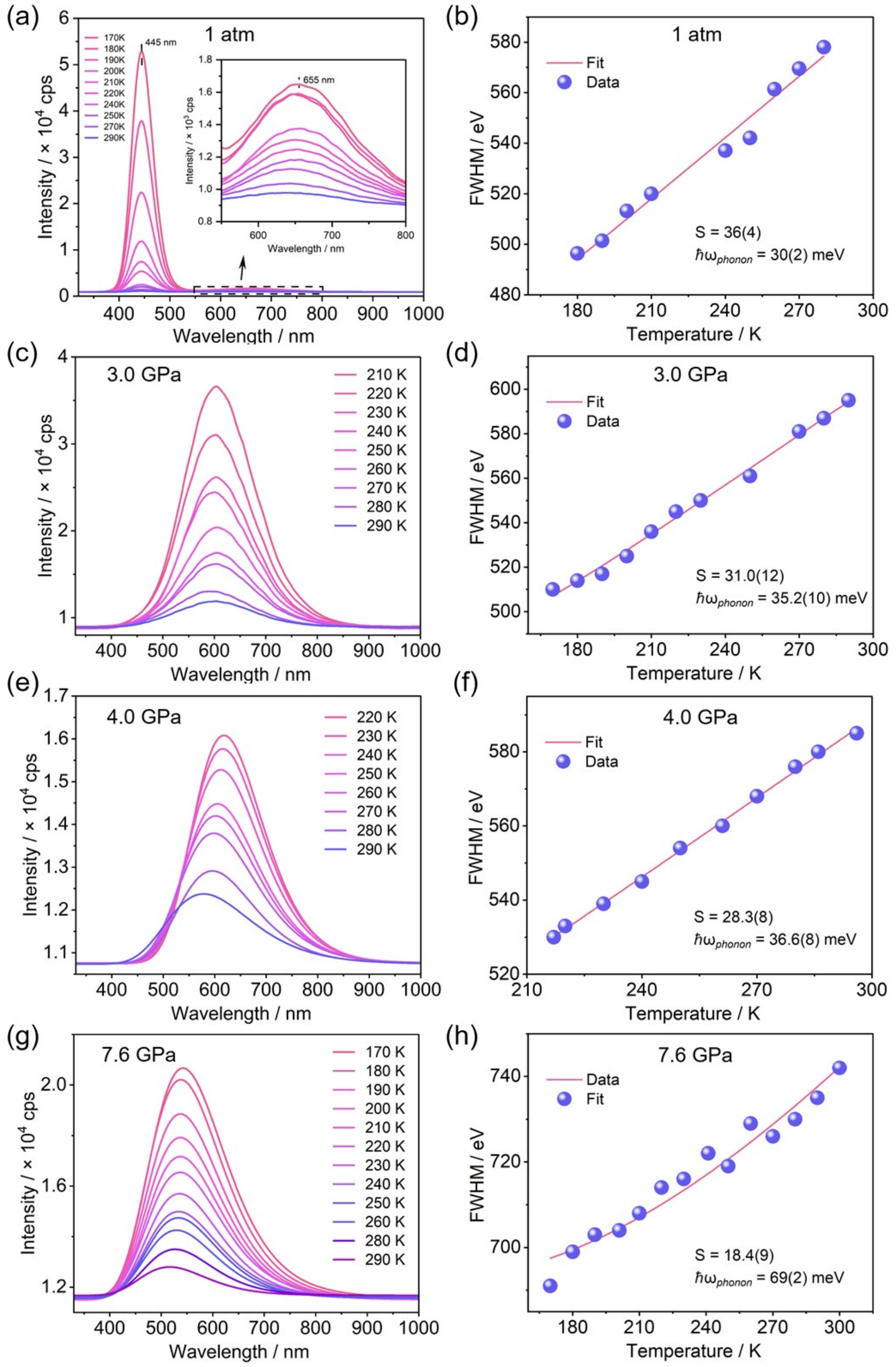

Figure S11. The temperature-dependent PL spectra and the temperature-dependent FWHM of PL emission of peak II of $(C_4H_8N)_2PbCl_4$ under 325 nm excitation: (a) (b) 1 atm, (c)(d) 3.0 GPa, (e)(f) 4.0 GPa, (g)(h) 7.6 GPa. The *S* factor is calculated based on the theory of Toyozawa.

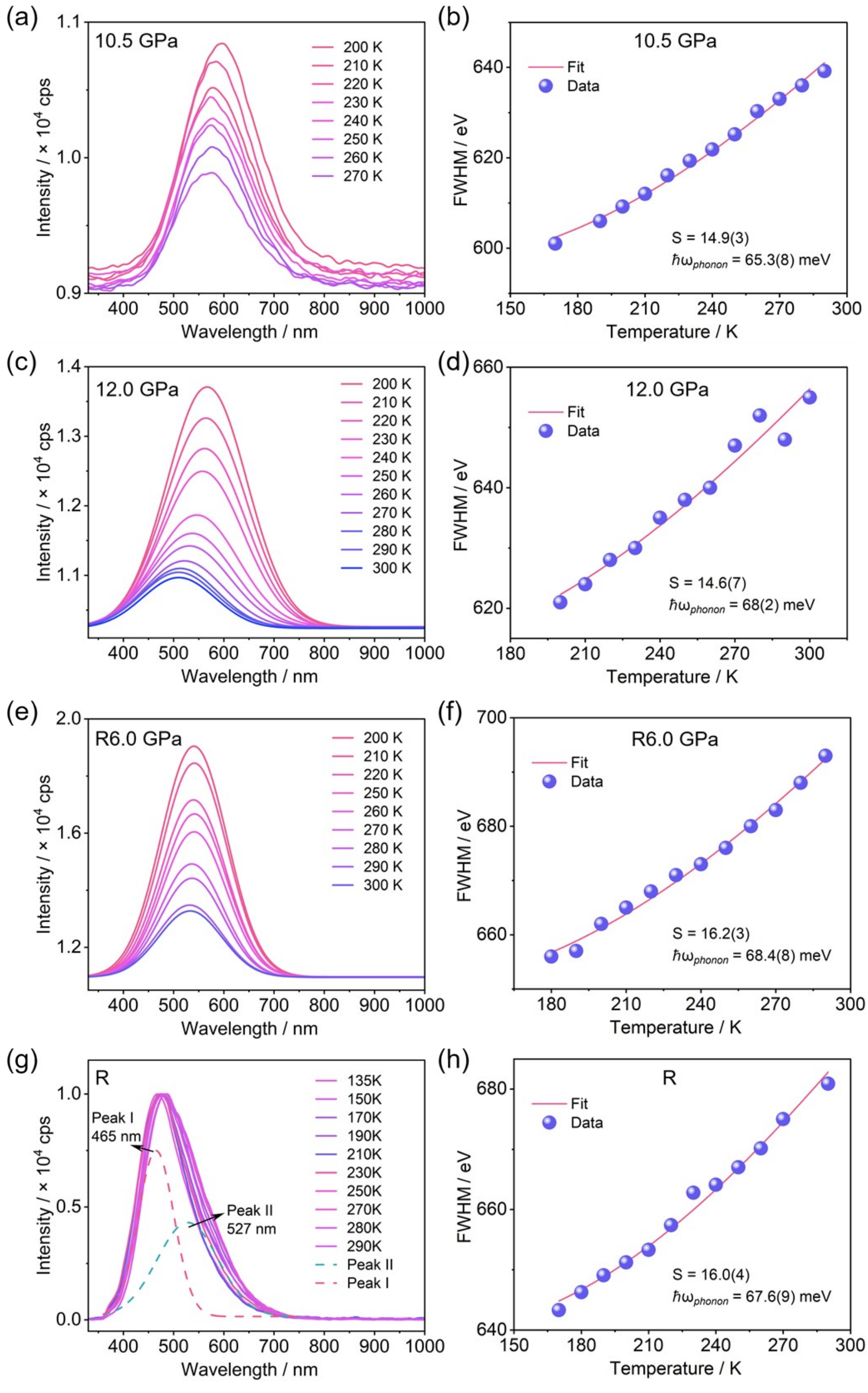

Figure S12. The temperature-dependent PL spectra and the temperature-dependent FWHM of PL emission of peak II of $(C_4H_8N)_2PbCl_4$ under 325 nm excitation: (a) (b) 10.5 GPa, (c)(d) 12.0 GPa, (e)(f) R6.0 GPa, (g)(h) Released. The *S* factor is calculated based on the theory of Toyozawa.

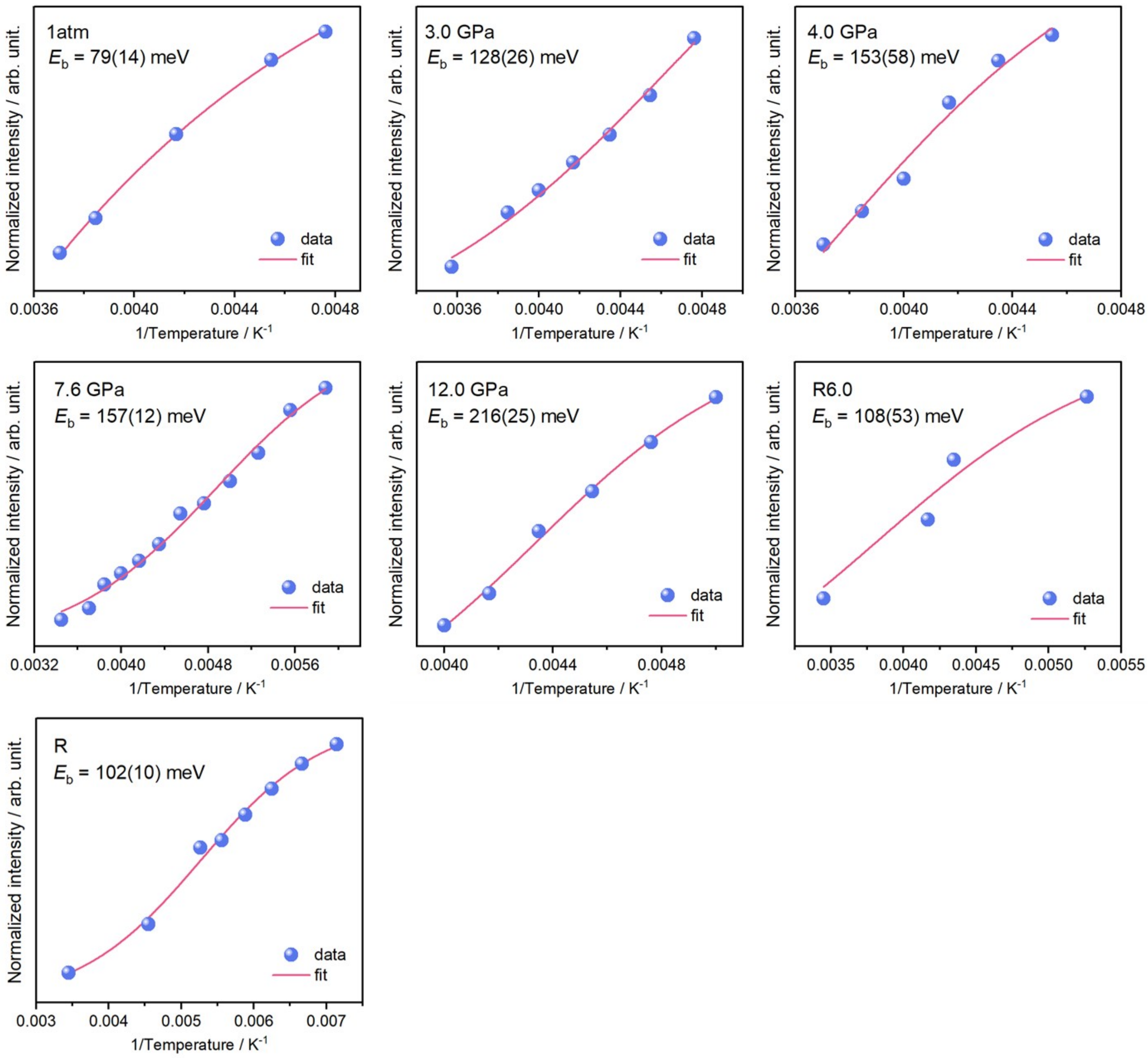

Figure S13. The binding energies of self-trapped excitons of $(C_4H_8N)_2PbCl_4$ measured at different pressures.

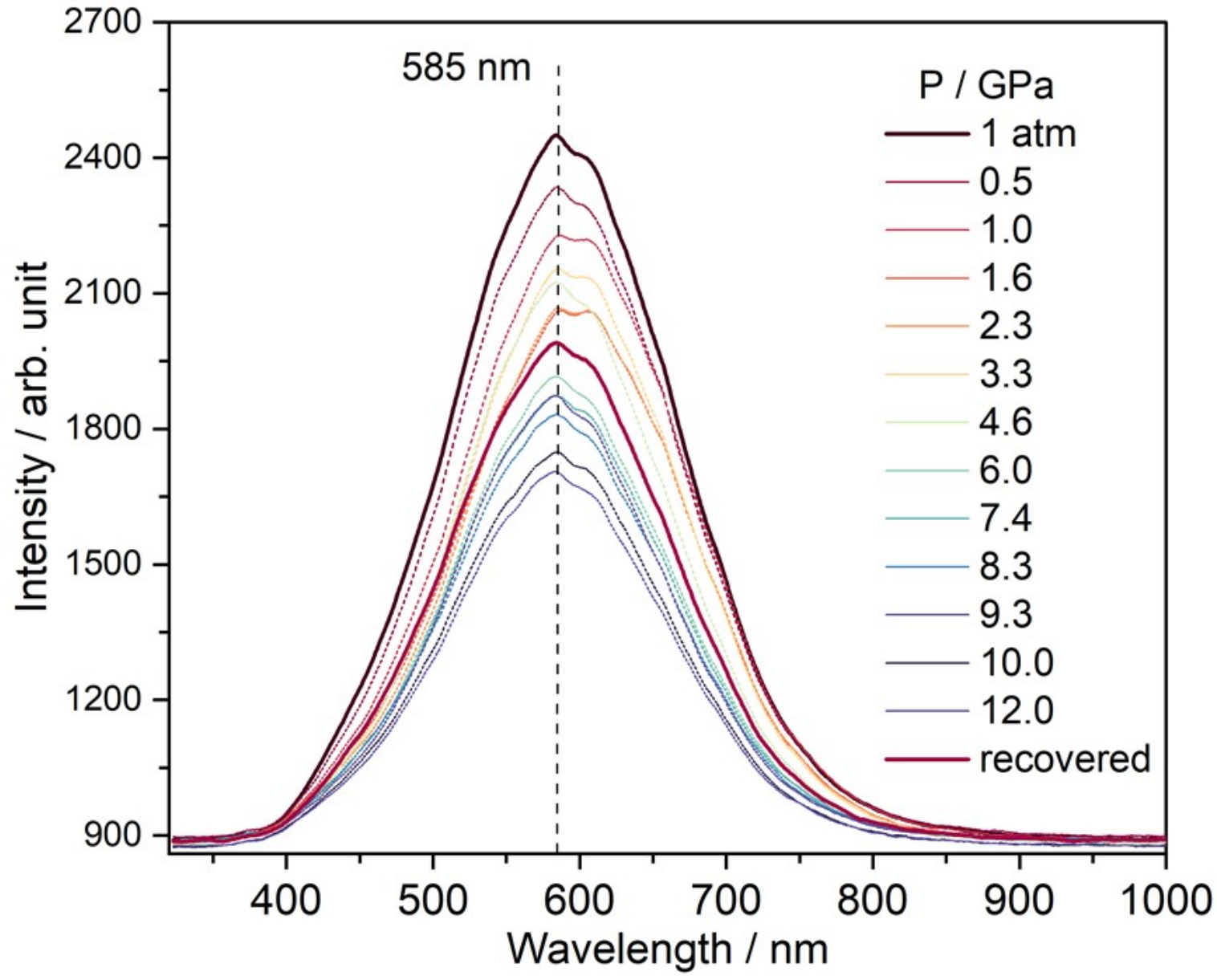

Figure S14. Pressure-dependent PL spectra of $C_4H_7N\cdot HCl$ crystal excited at 325 nm.

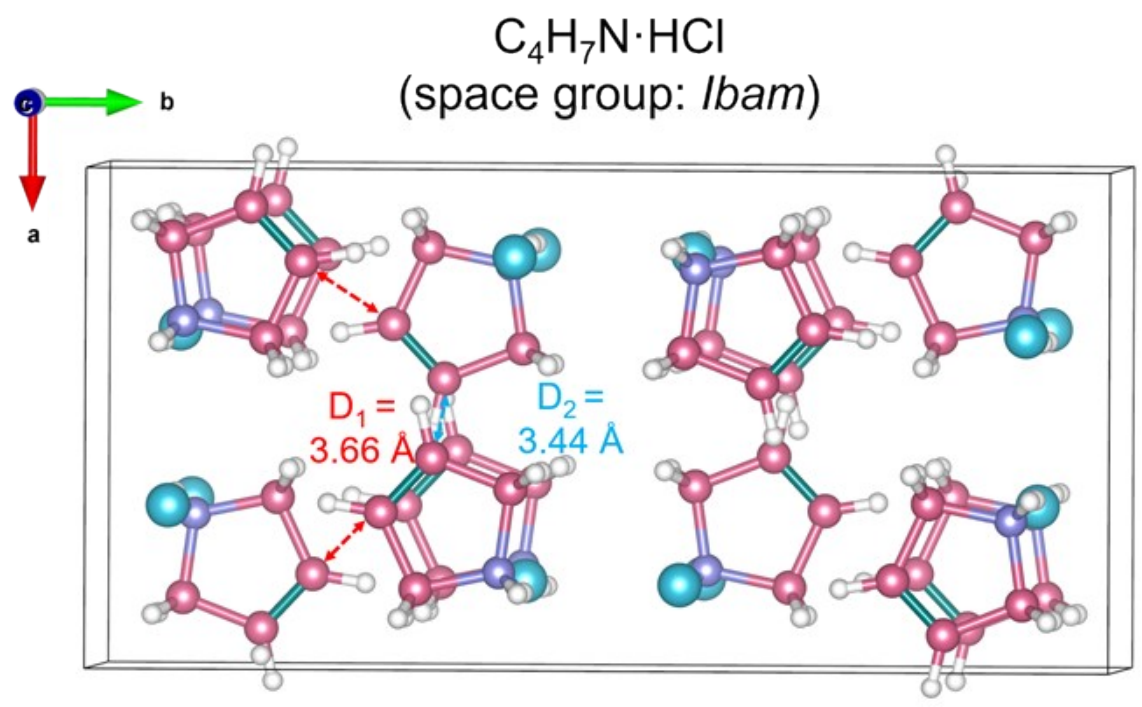

Figure S15. Crystal structure of the $C_4H_7N\cdot HCl$ projected along the *c*-axis. Pink, light blue, cyan, and white spheres denote C, N, Cl, and H atoms, respectively. The dashed arrows highlight the nearest neighbor distances between intermolecular sp^2 carbon atoms.

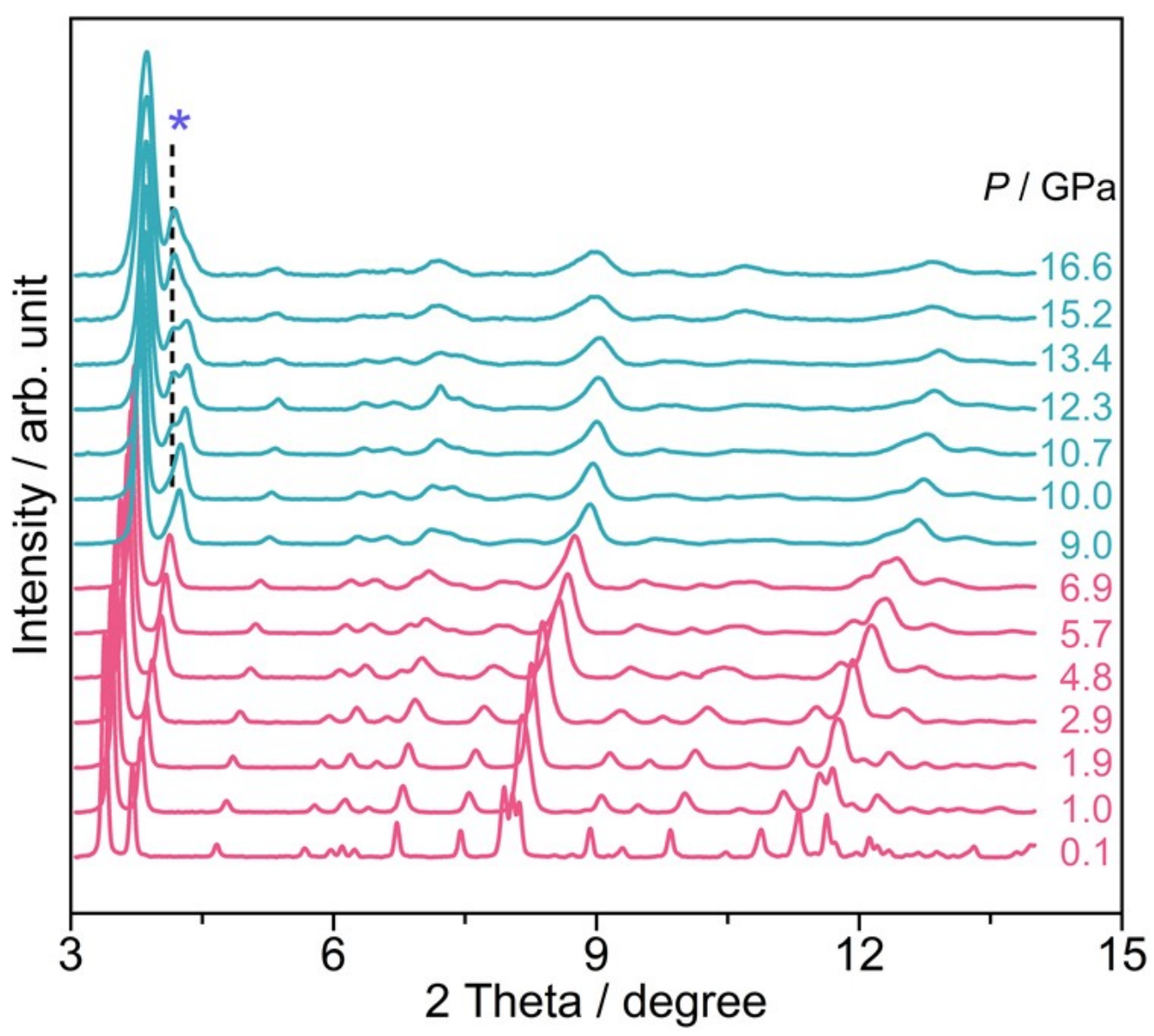

Figure S16. In situ synchrotron powder XRD patterns of $(C_4H_8N)_2PbCl_4$ under compression.

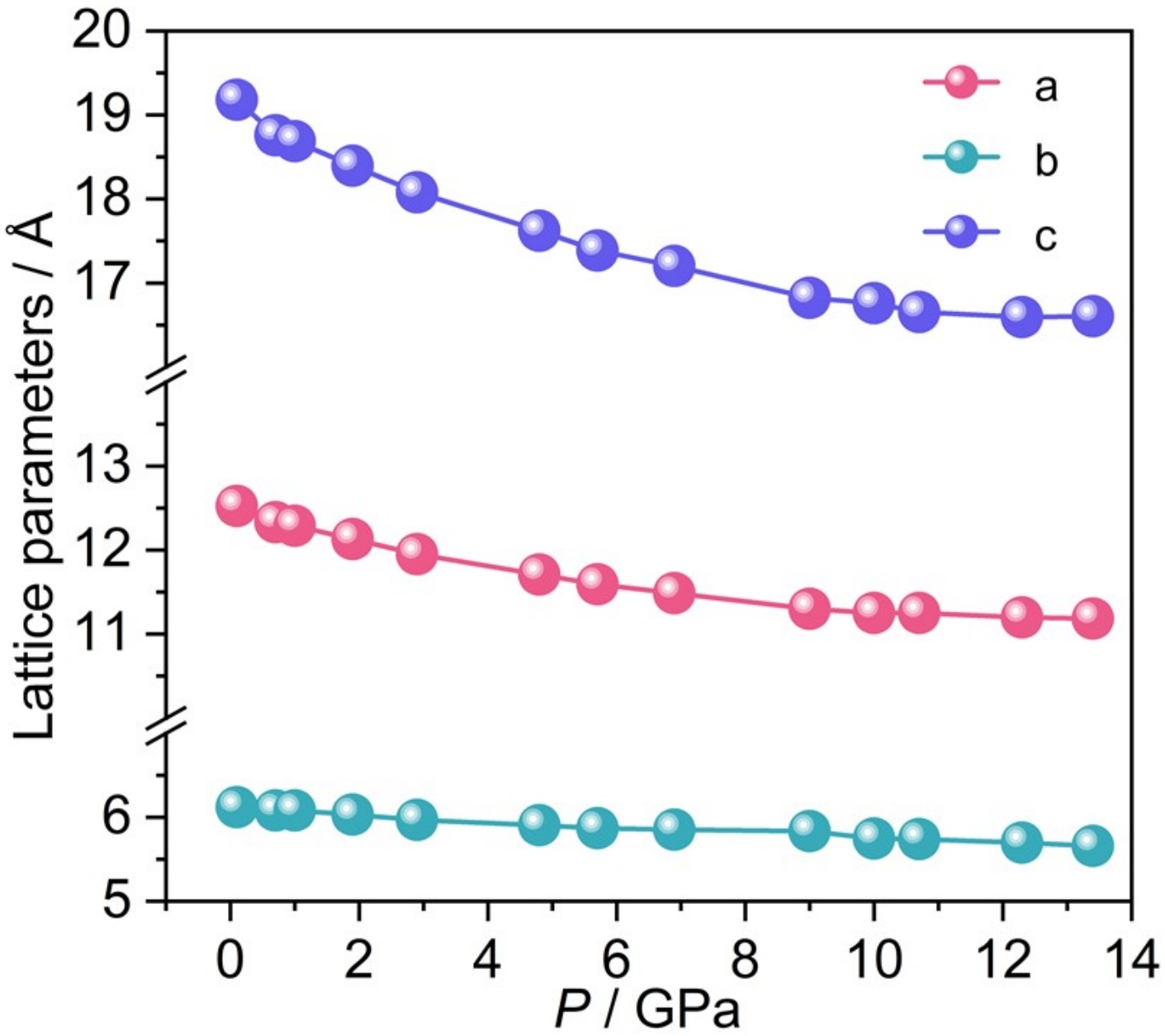

Figure S17. Evolution of lattice constants of $(C_4H_8N)_2PbCl_4$ under high pressure.

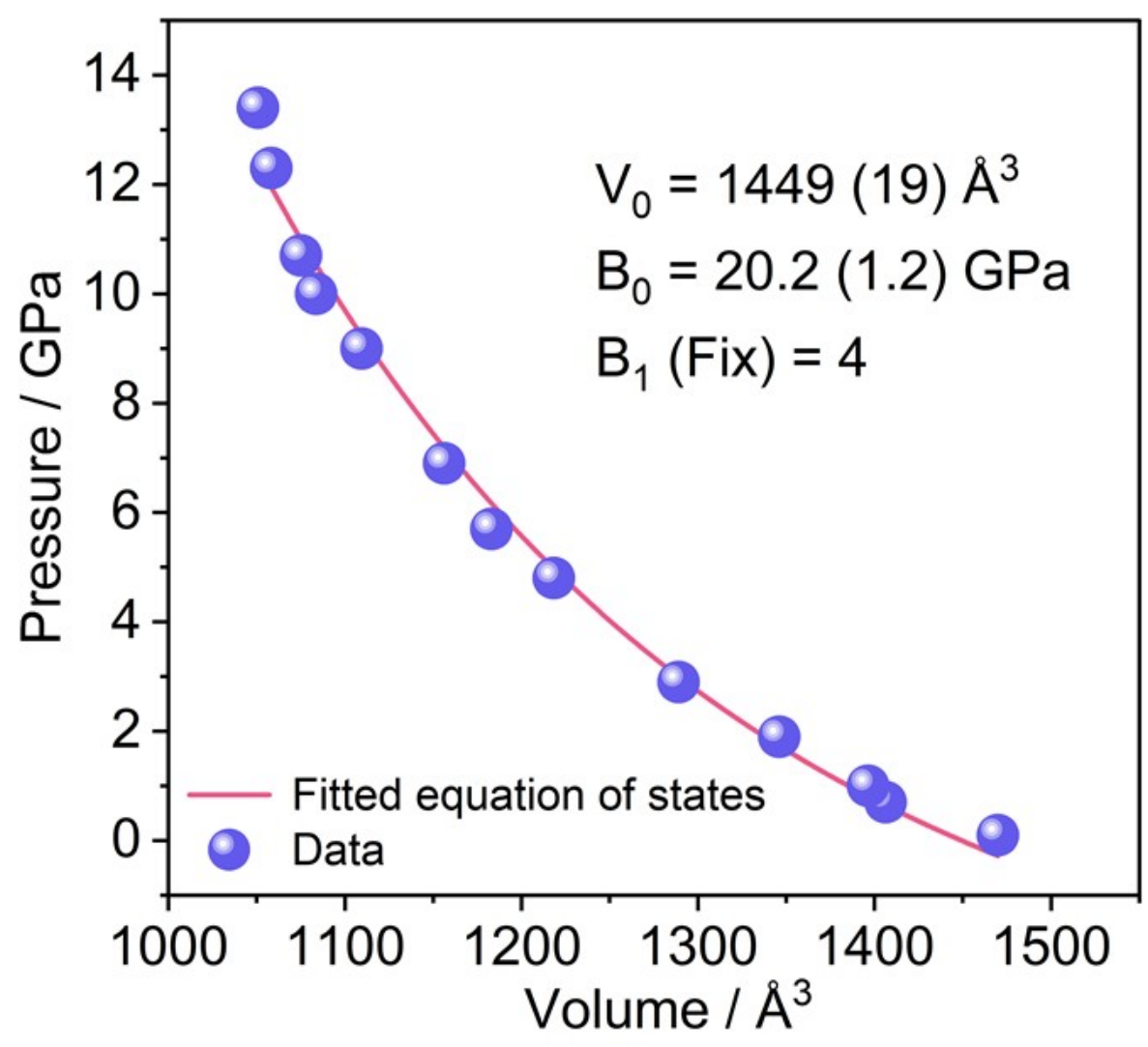

Figure S18. Unit cell volume of $(C_4H_8N)_2PbCl_4$ as a function of pressure.

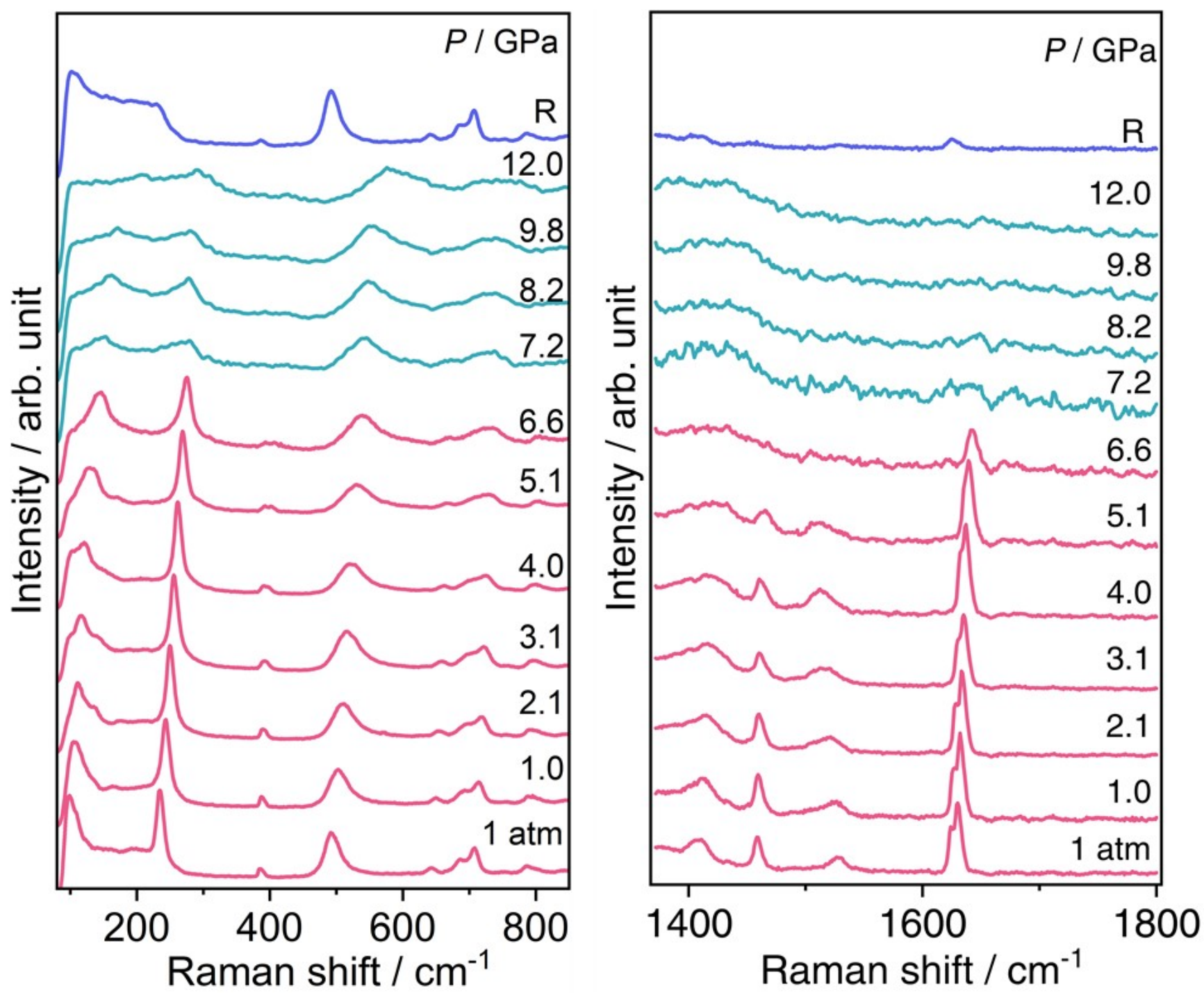

Figure S19. In situ Raman spectra of $(C_4H_8N)_2PbCl_4$ during pressure engineering.

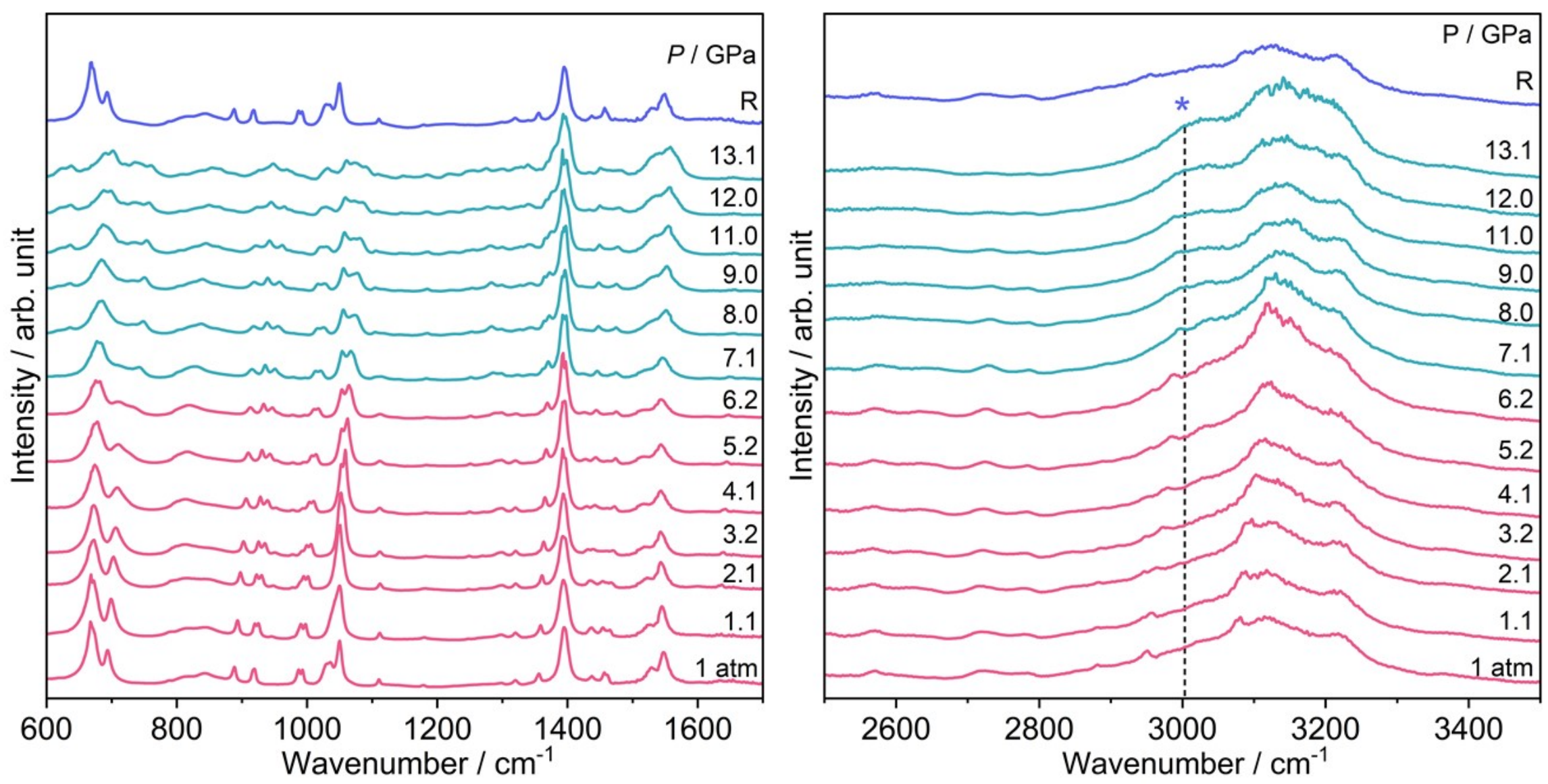

Figure S20. In situ IR spectra of $(C_4H_8N)_2PbCl_4$ during pressure engineering.

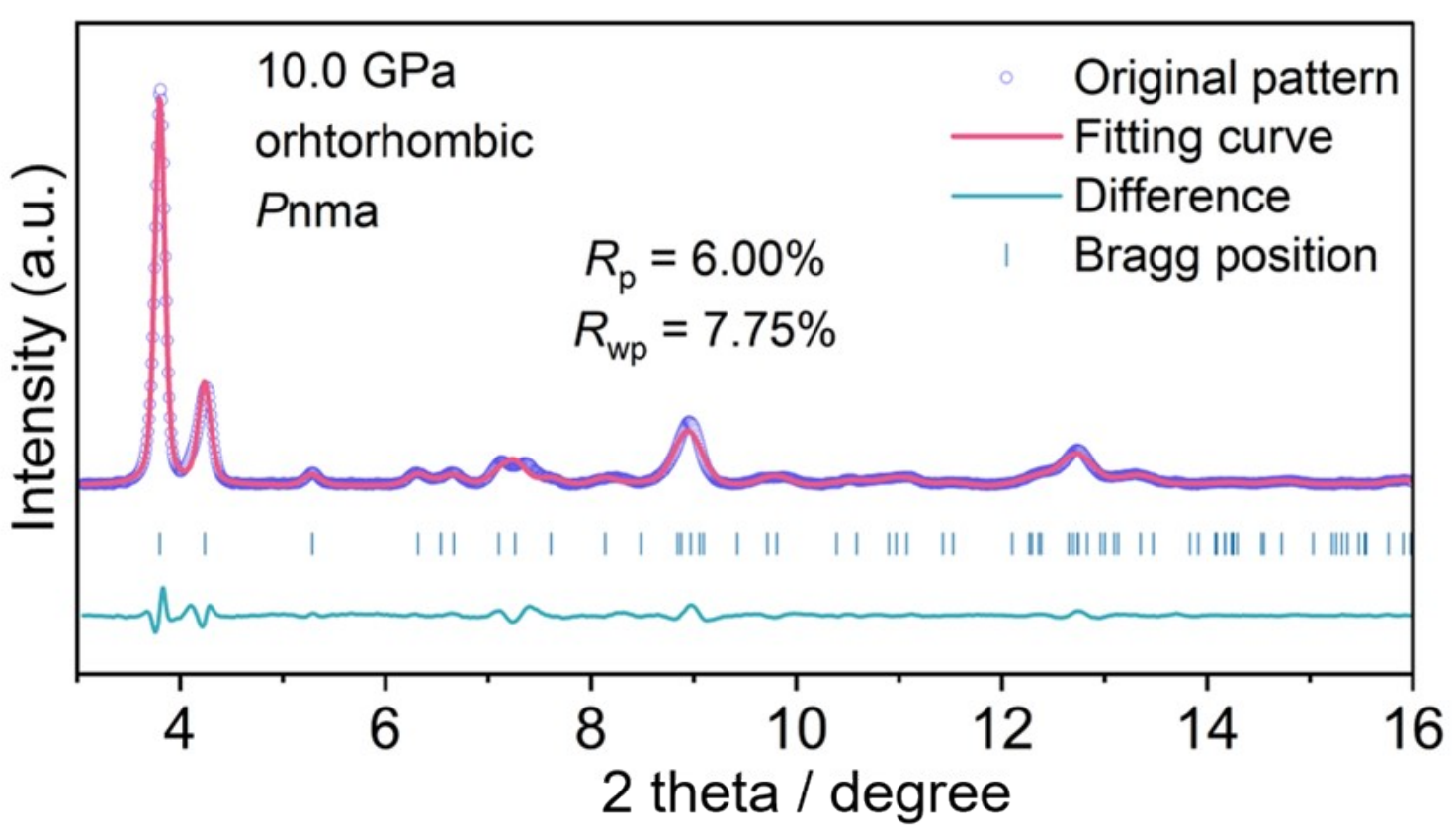

Figure S21. Rietveld refinement plot of $(C_4H_8N)_2PbCl_4$ under 10.0 GPa.

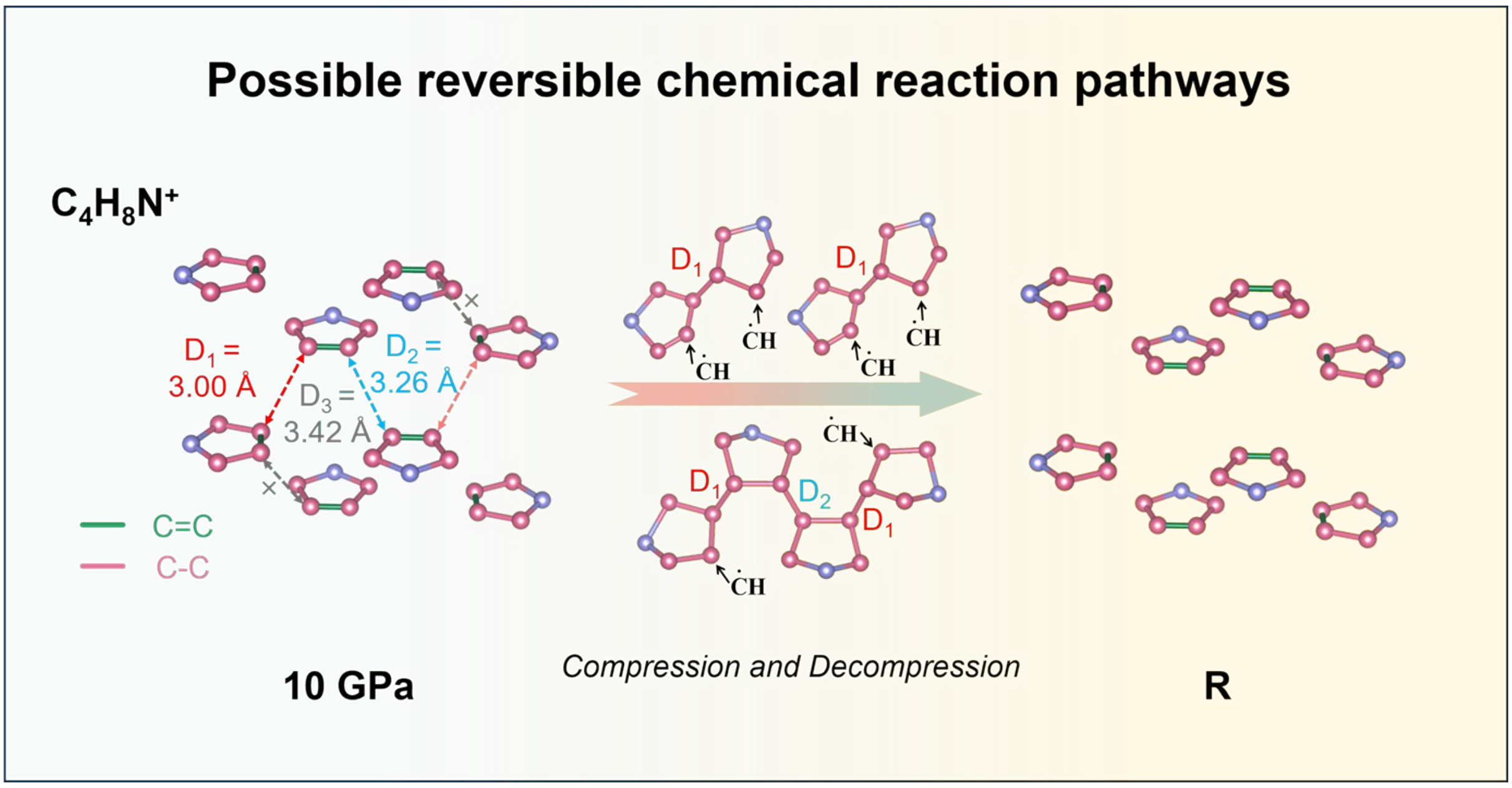

Figure S22. Possible reversible chemical reaction pathways.

Based on the crystal structure of $(C_4H_8N)_2PbCl_4$ at 10 GPa, the shortest intermolecular separation (D1) between sp^2 carbon atoms decreases to 3.00 Å. The reactivity of these carbon centers may lie between those of aromatic sp^2 carbons and alkyne sp carbons, for which critical intermolecular distances of approximately 2.8 and 3.1 Å, respectively, have been reported.[S1–S3] Thus, the D1 separation may be sufficiently short to permit pressure-induced C–C bond formation.

However, the inorganic $[PbCl_4]^{2-}$ framework partitions the organic cations into four isolated columns, confining possible reactions to quasi-one-dimensional channels. Although D2 (3.26 Å) may approach a reactive range upon further compression, D3 (3.42 Å) remains comparatively large, limiting further propagation. Consequently, bonding along D1, or possibly along a D1–D2–D1 sequence, would be expected to produce only short oligomers. Such species may retain unsaturated or reactive terminal sites, reducing their stability and making their recovery under ambient conditions unlikely.

References

[S1] T. C. Fitzgibbons, M. Guthrie, E. S. Xu, V. H. Crespi, S. K. Davidowski, G. D. Cody, N. Alem, J. V. Badding, *Nat. Mater.* 2015, **14**, 43-47.

[S2] J. Sun, X. Dong, Y. Wang, K. Li, H. Zheng, L. Wang, G. D. Cody, C. A. Tulk, J. J. Molaison, X. Lin, Y. Meng, C. Jin, H. K. Mao, *Angew. Chem. Int. Ed.* 2017, **56**, 6553-6557.

[S3] X. Tang, X. Dong, C. Zhang, K. Li, H. Zheng, H.-k. Mao, *Matter Radiat. Extremes* 2023, **8**, 058402.

Table S1. Crystal data and structure refinement for $(C_4H_8N)_2PbCl_4$.

Empirical formula	$(C_4H_8N)_2PbCl_4$
Identification code	mo_PbClC4N_0ma
CCDC	2337630
Empirical formula	$C_8H_{16}Cl_4N_2Pb$
Formula weight	489.22
Temperature/K	100.00
Crystal system	Orthorhombic
Space group	*Pnma*
a / Å	12.3270(8)
b / Å	6.0822(3)
c / Å	18.9439(11)
α / °	90
β / °	90
γ / °	90
Volume / $Å^3$	1420.32(14)
Z	4
ρ_{calc} / g·cm^{-3}	2.288
μ / mm^{-1}	12.604
F(000)	912.0
Crystal size / mm^3	0.1 × 0.1 × 0.08
Radiation	MoK_α (λ = 0.71073 Å)
2Θ range for data collection/°	5.424 to 50.054
Index ranges	$-14 \le h \le 14$, $-7 \le k \le 7$, $-22 \le l \le 22$
Reflections collected	16041
Independent reflections	1386 [R_{int} = 0.0442, R_{sigma} = 0.0201]
Data/restraints/parameters	1386/4/127
Goodness-of-fit on F^2	0.976
Final R indexes [I>=2σ (I)]	R_1 = 0.0179, wR_2 = 0.0445
Final R indexes [all data]	R_1 = 0.0202, wR_2 = 0.0459

$R_1 = \sum||F_o| - |F_c||/\sum|F_o|$. $wR_2 = [\sum w(F_o^2 - F_c^2)^2/\sum w(F_o^2)^2]^{1/2}$

Table S2. Bond lengths and bond angles for $[PbCl_4]^{2-}$ in $(C_4H_8N)_2PbCl_4$.

Bond	Length / Å	Bond	Angle / °
Pb-Cl1	2.9766	Cl1-Pb-Cl2	169.10
Pb-Cl2	2.7920	Cl1-Pb-Cl3	101.63
Pb-Cl3	2.6120	Cl1-Pb-Cl4	86.33
Pb-Cl4	3.2174	Cl1-Pb-Cl5	88.79
Pb-Cl5	3.0420	Cl1-Pb-Cl6	88.79
Pb-Cl6	3.0420	Cl2-Pb-Cl3	89.28
		Cl2-Pb-Cl4	82.77
		Cl2-Pb-Cl5	91.05
		Cl2-Pb-Cl6	91.05
		Cl3-Pb-Cl4	172.04
		Cl3-Pb-Cl5	90.95
		Cl3-Pb-Cl6	90.95
		Cl4-Pb-Cl5	89.20
		Cl4-Pb-Cl6	89.20
		Cl5-Pb-Cl6	177.19
